# Systematics of the *Rhinella margaritifera* complex (Anura, Bufonidae) from western Ecuador and Panama with insights in the biogeography of *Rhinella alata*

**DOI:** 10.3897/zookeys.501.8604

**Published:** 2015-04-30

**Authors:** Sueny P. dos Santos, Roberto Ibáñez, Santiago R. Ron

**Affiliations:** 1 Museo de Zoología, Departamento de Ciencias Biológicas, Pontificia Universidad Católica del Ecuador, Av. 12 de Octubre y Roca, Aptdo. 17–01–2184, Quito, Ecuador; 2 Smithsonian Tropical Research Institute, Apartado Postal 0843-03092, Panama, República de Panama; 3 Departamento de Zoología, Universidad de Panama, Panama, República de Panama

**Keywords:** Andes, Biogeography, Chocó, Morphology, Panama, Phylogeny, *Rhinella alata*

## Abstract

The *Rhinella margaritifera* species group consists of 17 species of toads distributed in tropical and subtropical South America and eastern Central America. The identity of some of its species is poorly understood and there are numerous undescribed cryptic species. Among them, the status of *Rhinella margaritifera* is one of the most problematic. Its range includes lowland rainforests separated by the Andes, the Chocoan rainforest to the west and the Amazonian rainforest to the east. This distribution is puzzling because the Andes are an old and formidable barrier to gene flow and therefore should generate vicariant speciation between disjunct lowland populations. Herein we clarify the taxonomy of populations of the *Rhinella margaritifera* complex from Central America and the Chocó region of South America. The morphological and genetic variation of *Rhinella margaritifera* was examined from 39 populations from Chocó, 24 from the upper Amazon region of Ecuador, and 37 from Panama, including the holotype of the Panamanian *Rhinella alata*. Phylogenetic analyses were performed based on mitochondrial genes 12S rRNA, 16S rRNA, and cytochrome c oxidase I (COI) and the nuclear gene Tyrosinase (Tyr). The genetic and morphological data show that Panamanian and Chocoan populations are conspecific. In the phylogeny, populations from Chocó and Panama form a well-supported clade. The morphology of the holotype of *Rhinella alata* falls within the variation range of Panamanian and Chocoan populations. Based on all this evidence, we assign the populations from western Ecuador and Panama to *Rhinella alata* and demonstrate that the unusual distribution pattern of “*Rhinella margaritifera*” on both sides of the Andes was an artifact of incorrectly defined species boundaries.

## Introduction

*Rhinella* is a genus of bufonid frogs distributed from southern Texas, through southern Sonora (Mexico), south tropical Mexico, Central America, and South America. There are 87 recognized species of *Rhinella* (Frost, 2014) among which 17 belong to the *Rhinella margaritifera* species group (Lavilla et al. 2013, Moravec et al. 2014). Thirteen of these species are distributed throughout the Amazon Basin, the Guyanas and Central America, while *Rhinella hoogmoedi* Caramaschi & Pombal, 2006 occurs in the Brazilian Atlantic Forest, *Rhinella scitula* (Caramaschi & Niemeyer, 2003) and *Rhinella ocellata* (Günther, 1858) in the Brazilian Cerrado, and *Rhinella paraguayensis* Ávila, Pansonato & Strüssmann, 2010 in the Brazilian Pantanal (Caramaschi and Niemeyer 2003, Caramaschi and Pombal 2006, Lima et al. 2007, Fouquet et al. 2007a, Ávila et al. 2010, Frost 2014). They inhabit the forest floor and their cryptic coloration mimics the forest leaflitter. Morphologically they have been characterized by the presence of hypertrophied supra and postorbital crests, especially in females. Putative synapomorphies for the group are the expansion of the posterior ramus of the pterygoid and nasals that articulate laterally with the preorbital process of the maxilla (Pramuk 2006).

The *Rhinella margaritifera* species group (formerly *Bufo typhonius* or *Bufo margaritifer* group) has one of the most complex histories in the systematics of Neotropical anurans (Hoogmoed 1986, 1989, 1990, Hass et al. 1995, Fouquet et al. 2007b). The boundaries among its species member are poorly understood as a result of a highly variable intraspecific morphology and scant morphological differentiation between some species. In addition, some of the type material is unavailable or poorly preserved and several species descriptions lack details. Despite recent progress in the systematics of the group (i.e. Vélez-Rodriguez 2004, Pramuk 2006, Fouquet et al. 2007b, 2012b, Ávila et al. 2010, Lavilla et al. 2013, Moravec et al. 2014) a number of cryptic species still need to be identified, specially among Amazonian populations (Hoogmoed 1990, Hass et al. 1995, Vélez-Rodríguez 2004, Pramuk 2006, Fouquet et al. 2007b, Lavilla et al. 2013, Moravec et al. 2014).

Two species of the *Rhinella margaritifera* group have been reported west of the Andes (Chocó region, humid forests west of the Andes in Colombia and Ecuador) and in eastern Panama: *Rhinella alata* and *Rhinella margaritifera*. *Rhinella alata* was described by Thominot (1884) as *Bufo alatus*, based on an adult male collected at Obispo, Isthmus of Panama. Boulenger (1885) considered it a junior synonym of “*Bufo typhonius*”, and Hoogmoed (1986, 1989) suggested that it was, possibly, a synonym of *Bufo acutirostris* (Spix, 1824). La Marca (1997) reported populations of *Rhinella alata* from northern Venezuela. Gorzula and Señaris (1999) suggested that *Rhinella margaritifera* only occurs in southern Venezuela and *Rhinella alata* north of the Orinoco. However, Barrio-Amorós (1999 “1998”, 2004) disagreed with both reports and considered that *Rhinella alata* was not distributed in Venezuela.

*Rhinella margaritifera* was described by Laurenti in 1768. It occurs in eastern Panama (Frost 2014), the Chocoan lowlands of western Ecuador and western Colombia (e.g. Anderson 1945, Miyata 1982, Ruiz-Carranza et al. 1996, Ortega-Andrade et al. 2010, Ortiz et al. 2013, Ron et al. 2014), Amazonia and vicinities in Bolivia, Brazil, Colombia, Ecuador, French Guiana, Guyana, Peru, Surinam and Venezuela (Lavilla et al. 2013). A genetic study by Fouquet et al. (2007b), using two mitochondrial genes (12S and 16S) and the two nuclear genes (Tyrosinase and 18S), showed that *Rhinella margaritifera* was paraphyletic and contained up to 11 cryptic species. Populations from the Chocó region have been widely referred as *Rhinella margaritifera* although Solis et al. (2010) remarked that populations from the Ecuadorian Chocó might belong to a separate species. Unfortunately, they did not provide further details.

The distribution of *Rhinella margaritifera* in the humid lowlands west and east of the Andes is intriguing because, particularly for amphibians, the Andes represent a formidable barrier to gene flow (e.g. Santos et al. 2009). Despite similar environmental conditions, only four amphibian species are shared between the lowland rainforests of the Amazon basin and the Chocó: *Rhinella margaritifera*, *Rhinella marina*, *Hypsiboas boans* and *Trachycephalus typhonius*. Moreover, there is genetic and morphological evidence suggesting that populations on each side of the Andes of *Rhinella marina* and *Trachycephalus typhonius* represent separate species (Slade and Moritz 1998, Ron and Read 2011). Thus, the distribution of *Rhinella margaritifera* is suggestive of either an unusual biogeographic history or the existence of cryptic species.

Herein, genetic and morphological information were integrated to clarify the taxonomy of the populations of *Rhinella margaritifera* from Panama and the Chocoan region. Populations from the western and eastern Andean slopes were compared to test the role of the Andes as a dispersal barrier in shaping the evolution of the *Rhinella margaritifera* species complex.

## Methods

### Population sampling

**Figure 1.**
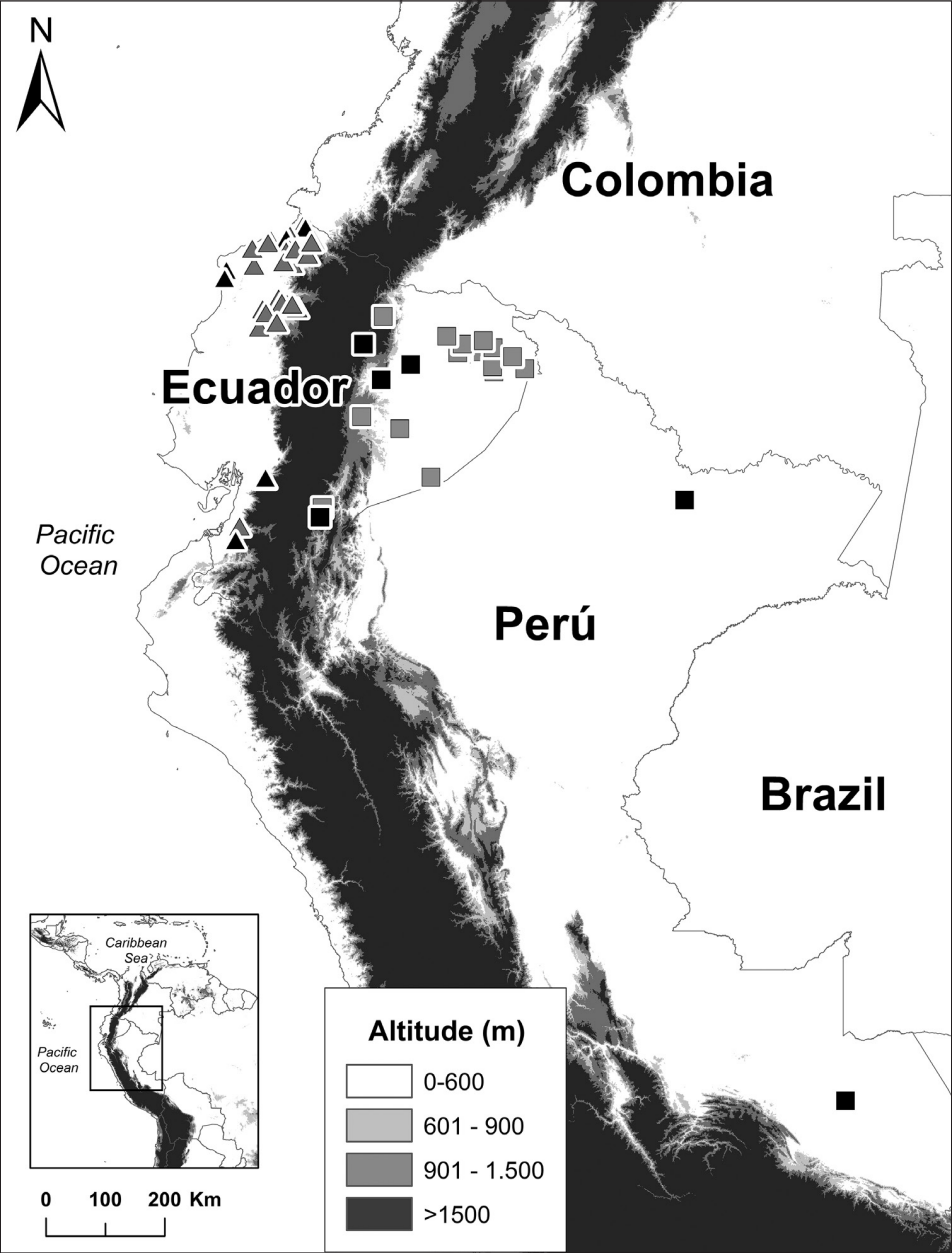
Localities of the *Rhinella margaritifera* group from Chocó (triangles) and Amazon (squares). Gray for specimens analyzed morphologically, black for specimens analyzed both genetically and morphologically. Specimens (listed in Appendix 1 and Table 1) are deposited at the Museo de Zoología of Pontificia Universidad Católica del Ecuador (QCAZ), Centro de Ornitología y Biodiversidad (CORBIDI), and National Museum of Natural History (USNM).

**Figure 2.**
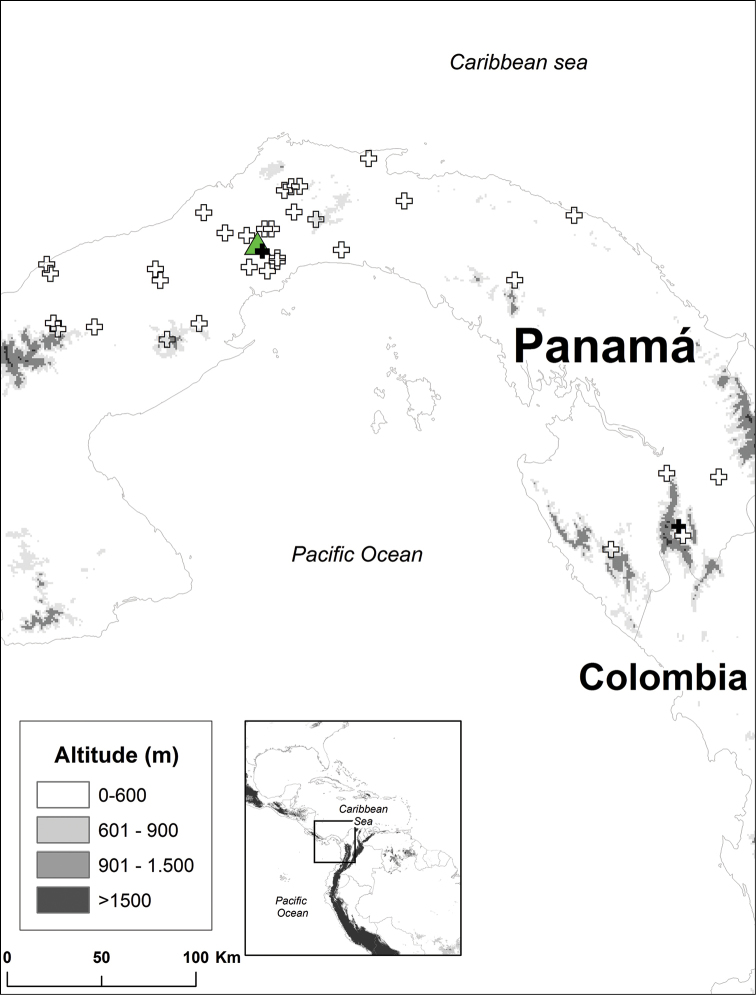
Panamanian populations of the *Rhinella margaritifera* group included in this study. White crosses for specimens analyzed morphologically, black crosses analyzed both morphologically and genetically. The type locality of *Rhinella alata* is shown with a triangle. Specimens (listed in Appendix 1 and Table 1) are deposited at American Museum of Natural History (AMNH), Muséum National d’Histoire Naturelle du Paris (MNHN), Círculo Herpetológico de Panama (CH), and the Museo de Vertebrados de la Universidad de Panama (MVUP).

Populations from Panama, the Ecuadorian Chocó, and the Amazon basin were sampled (Figs 1 and 2). Specimens examined morphologically are listed in Appendix 1; specimens analyzed genetically are listed in Table 1.

Morphometric analyses were based on 120 adult specimens of *Rhinella margaritifera* from Panama (14 specimens from 10 populations), Ecuadorian Chocó (74 specimens, 37 populations), and the Ecuadorian Amazon (32 specimens, 18 populations). Qualitative morphological characters were examined in the same specimens and 28 additional individuals from 27 Panamanian populations (Figs 1 and 2; Appendix 1).

Genetic analyses were based on newly generated sequences of *Rhinella margaritifera* from 32 individuals and 19 populations: *Rhinella margaritifera* from the Ecuadorian Chocó (12 individuals, 7 populations); *Rhinella margaritifera* from Panama (3 individuals, 2 populations) and *Rhinella margaritifera* from the Amazon basin (17 individuals, 10 populations), and six sequences for the outgroups (see Table 1). Sequences of eight *Rhinella dapsilis* were generated, including all available homologous sequences for the *Rhinella margaritifera* species group from GenBank (http://www.ncbi.nlm.nih.gov/genbank; Table 1). *Rhinella marina*, *Rhinella chavin*, *Rhinella nesiotes* and *Rhinella festae* were included as outgroups. The morphometric and genetic analyses were based on the same individuals, when possible. Several specimens used in the morphological analyses lacked tissues and were not included in the genetic analyses. However, their identification was unambiguous based on geographic distribution and morphological characters.

Examined specimens are deposited at the Museo de Zoología, Pontificia Universidad Católica del Ecuador (QCAZ, Quito, Ecuador), the American Museum of Natural History (AMNH, New York, USA), Círculo Herpetológico de Panama (CH, Panama, Panama), Centro de Ornitología y Biodiversidad (CORBIDI, Lima, Perú) and Museo de Vertebrados de la Universidad de Panama (MVUP). We also examined photographs of the holotypes of *Rhinella alata* from Musée National d’Historie Naturelle (MNHN, Paris, France). Tissues were obtained from the QCAZ and CH collections. Tissues (liver or thigh muscle) were stored in 95% ethanol.

### Morphological analyses

Morphological terminology and abbreviations follow Vélez-Rodriguez (2004) and Narvaes and Rodrigues (2009). Sexual maturity was determined by the presence of nuptial pads in adult males and convoluted oviducts or mature eggs in gravid females. Specimens from the QCAZ collection were euthanized with the anesthetic spray Roxicaine, fixed in 10% formalin, and preserved in 70% ethanol.

The goal of the morphological analyses was to compare three geographic regions: (1) Chocó (2) Panama, and (3) upper Amazon basin. Because the phylogeny showed that Panama and Chocó populations are conspecific, we also compared Chocó + Panama vs. upper Amazon. Morphometric analyses were based on adult and well-preserved specimens (Simmons 2002). We measured the following variables: (1) SVL (snout-vent length, from the tip of snout to the mid-vent); (2) TL (tibia length, from the outer edge of flexed knee to the heel); (3) FL (femur length, from the mid-venter to the outer edge of flexed knee); (4) HL (head length, from the posterior margin of tympanum to the tip of snout); (5) HW (head width, between knobs at angles of jaws, if present); (6) STCH (supratympanic crest height, the distance between the angle of the jaw and the highest point of the ridge above of the tympanum); (7) SOCH (supraorbital crest height, the distance between the angle of jaw and the highest point of the ridge at the mid-orbit); (8) NSD (nostril-snout distance, from the nostril to the tip of the snout); (9) IND (inter-nostril distance, distance between nostrils); (10) TD (tympanum diameter, from the posterior to the anterior edge of the tympanum); (11) FT (foot length, from the posterior edge of the metatarsal tubercle to the tip of the toe IV). Measurements were taken with digital calipers (to the nearest 0.01 mm). Two qualitative morphological characters were also analyzed: (1) vertebral apophyses (present/absent) and (2) bony knob at angle of jaws (present/absent).

Principal Components Analysis (PCA) and Discriminant Function Analysis (DFA) were used to assess morphometric differentiation between Chocó, upper Amazon, and Panama. To remove the effect of body size (SVL), the MANOVA and PCA were applied to the residuals from the linear regressions between the measured variables and SVL, for males and females separately. For the PCA, only components with eigenvalues > 1 were retained. All measurements were first subjected to the Shapiro-Wilk normality to test for normal distribution (Shapiro and Wilk 1965). Data not normally distributed were log-transformed. Levene’s test was used to determine if variables were homoscedastic (Levene 1960). Number of analyzed specimens were (1) Chocó: 43 males and 31 females, (2) Panama: 6 males and 8 females, (3) upper Amazon basin: 16 males and 16 females. All analyses were performed using JMP® 9.0.1 (SAS Institute 2010).

### DNA extraction, amplification, and sequencing

Total DNA was extracted from muscle or liver tissue preserved in 95% ethanol or tissue storage buffer using standard guanidine thiocyanate protocol (M. Fujita, unpublished) with modifications. Polymerase Chain Reaction (PCR) was used to amplify the mitochondrial genes 12S rRNA, 16S rRNA, cytochrome c oxidase I (COI) and nuclear gene Tyrosinase (Tyr). PCR amplifications were carried out under standard protocols. Using standard primers developed by Bossuyt and Milinkovitch (2000), Goebel et al. (1999), Pauly et al. (2004), and Meyer et al. (2005). Amplicons were sequenced by Macrogen Inc., Seoul, Korea.

### Phylogenetic analyses and genetic distances

Preliminary sequence alignment was done with Geneious Pro 5.4.6 (Drummond et al. 2011). The sequence matrix was imported to Mesquite 2.75 (Maddison and Maddison 2011) and the ambiguously aligned regions were adjusted manually to produce a parsimonious alignment. Phylogenetic trees were obtained using Bayesian Inference (BI) in MrBayes 3.1.2 (Ronquist and Huelsenbeck 2003) and Maximum Likelihood (ML) in Garli 2.0 (Zwickl 2006). The best-fit models of sequence evolution were selected under the Akaike information criterion (AIC) and the best partitioning scheme for the combined nucleotide data set and the models of character evolution for the BI and ML were estimated with PartitionFinder 1.0.1 (Lanfear et al. 2012). We ran three analyses: (1) the complete multi-locus data set, (2) only mitochondrial genes, (3) only the nuclear gene.

The Bayesian search consisted of two parallel runs each with 130 × 10^6^ generations with four Markov chains. The convergence of the runs was assessed with Tracer 1.5 (Rambaut and Drummond 2007) evaluating the effective sample sizes and stopping when all post burn-in values were greater than 200. The first 10% of the sample was discarded as burn-in (Castañeda and Queiroz 2011).

For the ML analysis, we carried out 20 replicate searches and increased the setting “genthreshfortopoterm” until all searches resulted in similar likelihood values, indicating an efficient search (Zwickl 2006; final value was 200,000). Ten replicate searches started from stepwise trees and ten from random trees. The setting “limsprrange” was set to 10 (default = 6). Node support was assessed with non-parametric bootstrapping (Felsenstein 1983) with 100 pseudoreplicates with the same settings of the stepwise full search but with a single replicate per search. The 50% majority rule consensus for the bootstrap trees was obtained with Mesquite 2.75 (Maddison and Maddison 2011).

Uncorrected pairwise (*p*) genetic distances were obtained for gene *16S* using software Mesquite 2.75 (Maddison and Maddison 2011). Missing and ambiguous sites were excluded. Genetic distances comparisons were based on gene *16S* because it has been widely used as a barcode standard in amphibians (e.g. Vences et al. 2005). We assumed that genetic distances > 3% are suggestive of interspecific differentiation (Fouquet et al. 2007c). Genetic distances thresholds are problematic because they can lead to both false negatives and false positives in species identifications (Collins and Cruickshank 2013). We used the threshold only as a working hypothesis that was tested with morphological comparisons.

**Table 1.** GenBank accession numbers for DNA sequences used in the phylogenetic analysis.

Museum No.	Species	Country	Locality	GenBank Accession No.				Reference
				TYR	16S	12S	COI	
QCAZ10253	*Rhinella alata*	Ecuador	Reserva La Chiquita	KR012523	KR012615	KR012605	KR012568	This study
QCAZ10254	*Rhinella alata*	Ecuador	Reserva La Chiquita	KR012524	KR012616	KR012601	KR012567	This study
QCAZ10255	*Rhinella alata*	Ecuador	Reserva La Chiquita	KR012525	KR012617	KR012602	KR012570	This study
QCAZ11598	*Rhinella alata*	Ecuador	Reserva La Chiquita	KR012526	KR012618	KR012603	KR012550	This study
QCAZ13882	*Rhinella alata*	Ecuador	Manta Real	KR012527	KR012619	KR012597	KR012571	This study
QCAZ13896	*Rhinella alata*	Ecuador	Manta Real	-	DQ158471	DQ158471	-	Pramuk 2006
QCAZ14607	*Rhinella alata*	Ecuador	Borbón	KR012528	KR012620	KR012578	KR012552	This study
QCAZ37244	*Rhinella alata*	Ecuador	Valle Hermoso	KR012539	KR012632	KR012592	KR012576	This study
QCAZ37248	*Rhinella alata*	Ecuador	Valle Hermoso	KR012540	KR012633	KR012595	KR012544	This study
QCAZ 23161	*Rhinella alata*	Ecuador	San Lorenzo	KR012534	KR012626	KR012577	KR012562	This study
QCAZ25023	*Rhinella alata*	Ecuador	La Tortuga	KR012536	KR012629	KR012596	KR012572	This study
QCAZ25025	*Rhinella alata*	Ecuador	La Tortuga	KR012537	KR012630	KR012582	KR012573	This study
QCAZ25032	*Rhinella alata*	Ecuador	La Pedorrera	KR012538	KR012631	KR012604	KR012569	This study
CH9104	*Rhinella alata*	Panama	Cana, Boca Cupé	KR012507	KR012610	KR012598	KR012560	This study
MVUP2299	*Rhinella alata*	Panama	Río Chico Masambí, Parque Nacional Soberanía	KR012511	KR012613	KR012600	KR012561	This study
CH9192	*Rhinella alata*	Panama	Parque Nacional Soberanía	KR012521	KR012611	KR012599	KR012559	This study
QCAZ11597	*Rhinella alata*	Ecuador	Reserva La Chiquita	-	DQ15872	DQ15872	-	Pramuk 2006
104mc	*Rhinella castaneotica*	French Guyana	Tibourou	EF364355	EF364289	EF364263	-	Fouquet et al. 2007b
110pg	*Rhinella castaneotica*	French Guyana	Moint Saint Marcel	EF364353	EF364285	EF364259	-	Fouquet et al. 2007b
QCAZ38477	*Rhinella dapsilis*	Ecuador	Villano B	KR012513	KR012634	KR012586	KR012554	This study
QCAZ38512	*Rhinella dapsilis*	Ecuador	Villano BII	KR012514	KR012635	KR012587	KR012558	This study
QCAZ38560	*Rhinella dapsilis*	Ecuador	Villano B	KR012515	KR012636	KR012588	KR012555	This study
QCAZ38621	*Rhinella dapsilis*	Ecuador	Villano K4	KR012516	KR012637	KR012606	KR012556	This study
QCAZ38688	*Rhinella dapsilis*	Ecuador	Villano K4	KR012517	KR012638	KR012607	KR012575	This study
QCAZ38755	*Rhinella dapsilis*	Ecuador	Villano BII	KR012518	KR012639	KR012589	KR012548	This study
QCAZ38892	*Rhinella dapsilis*	Ecuador	Comunidad Kutintza 2	KR012519	KR012640	KR012608	KR012566	This study
QCAZ38998	*Rhinella dapsilis*	Ecuador	Comunidad Kurintza 3	KR012520	KR012641	KR012590	KR012549	This study
MTR19199	*Rhinella hoogmoedi*	Brazil	Bahia, Camacan	-	JN867571	JN867545	-	Fouquet et al. 2012a
112bm	*Rhinella lescurei*	French Guyana	Litany	EF364343	EF217473	EF364279	-	Fouquet et al. 2007b
3027t	*Rhinella lescurei*	French Guyana	Mitaraka	JN692065	EF364305	EF364279	-	Fouquet et al. 2012b
108mc	*Rhinella margaritifera*	French Guyana	Kaw	EF364333	EF364292	EF364266	-	Fouquet et al. 2007b
136mc	*Rhinella margaritifera*	French Guyana	Crique Margot	EF364335	EF364292	EF364266	-	Fouquet et al. 2007b
389MC	*Rhinella margaritifera*	French Guyana	Camp Canopé	JN692029	-	-	-	Fouquet et al. 2007b
374MC	*Rhinella margaritifera*	French Guyana	Régina	JN692038	JN691389	JN690782	-	Fouquet et al. 2007b
390MC	*Rhinella margaritifera*	French Guyana	St Georges	JN692037	JN691388	JN690781	-	Fouquet et al. 2007b
2559T	*Rhinella margaritifera*	French Guyana	Pic Matecho	JN690780	JN691387	JN690780	-	Fouquet et al. 2012b
4482T	*Rhinella margaritifera*	French Guyana	Angoulème	JN692042	JN691379	JN690772	-	Fouquet et al. 2012b
163bm	*Rhinella margaritifera*	French Guayana	Guatemala	EF364320	EF364292	EF364266	-	Fouquet et al. 2007b
164bm	*Rhinella margaritifera*	French Guyana	Montagne des Singes	EF364321	EF364292	EF364266	-	Fouquet et al. 2007b
176bm	*Rhinella margaritifera*	French Guyana	Crique Grand Leblond	EF364323	EF364292	EF364266	-	Fouquet et al. 2007b
195mc	*Rhinella margaritifera*	French Guyana	Kaw	EF364325	EF364292	EF364266	-	Fouquet et al. 2007b
2034at	*Rhinella margaritifera*	French Guyana	Nouragues	JN692033	EF364292	EF364266	-	Fouquet et al. 2007b
204mc	*Rhinella margaritifera*	French Guyana	Saul	EF364328	EF364295	EF364269	-	Fouquet et al. 2007b
217mc	*Rhinella margaritifera*	French Guyana	Grant Santi	EF364329	EF364299	EF364273	-	Fouquet et al. 2007b
225mc	*Rhinella margaritifera*	French Guyana	Road St. Elie	EF364330	EF364292	EF364266	-	Fouquet et al. 2007b
284mc	*Rhinella margaritifera*	French Guyana	St Elie	EF364336	EF364292	EF364266	-	Fouquet et al. 2007b
288ag	*Rhinella margaritifera*	French Guyana	St Georges	JN692021	JN691380	JN690773	-	Fouquet et al. 2012b
294mc	*Rhinella margaritifera*	French Guyana	Camp Canope	JN692029	EF364292	EF364266	-	Fouquet et al. 2012b
2bm	*Rhinella margaritifera*	French Guyana	Cisame	EF364313	EF364293	EF364267	-	Fouquet et al. 2007b
307pg	*Rhinella margaritifera*	French Guyana	Lac Toponowini	JN692022	EF364292	EF364266	-	Fouquet et al. 2012b
361mc	*Rhinella margaritifera*	French Guyana	Lucifer	JN692031	EF364292	EF364266	-	Fouquet et al. 2012b
408pg	*Rhinella margaritifera*	French Guyana	Mont Kotika	JN692023	EF364292	EF364266	-	Fouquet et al. 2012b
66mc	*Rhinella margaritifera*	French Guyana	Monts Bakra	EF364334	EF364298	EF364272	-	Fouquet et al. 2007b
74af	*Rhinella margaritifera*	French Guyana	St Georges	JN692020	EF364266	EF364292	-	Fouquet et al. 2012b
92bm	*Rhinella margaritifera*	French Guyana	Cisame	EF364314	EF364301	EF364275	-	Fouquet et al. 2007b
KU215143	*Rhinella margaritifera*	Peru	Madre de Dios	-	AY819461	AY819331	-	Wiens et al. 2005
13872MTR	*Rhinella margaritifera*	Brazil	Amapá, Lourenço	JN692016	JN691390	JN690783	-	Fouquet et al. 2012b
13873MTR	*Rhinella margaritifera*	Brazil	Amapá, Lourenço	JN692017	JN691391	JN690784	-	Fouquet et al. 2012b
13874MTR	*Rhinella margaritifera*	Brazil	Amapá, Lourenço	JN692018	JN691393	JN690786	-	Fouquet et al. 2012b
13878MTR	*Rhinella margaritifera*	Brazil	Amapá, Lourenço	JN692019	JN691392	JN690785	-	Fouquet et al. 2012b
MRT6313	*Rhinella margaritifera*	Brazil	Pará, Serra do Kukoinhokren	JN692075	JN691394	JN690787	-	Fouquet et al. 2012b
MRT6317	*Rhinella margaritifera*	Brazil	Pará, Serra do Kukoinhokren	JN692076	JN691395	JN690788	-	Fouquet et al. 2012b
KU215146	*Rhinella margaritifera*	Peru	Madre de Dios	-	-	HM563858	JN867978	Mendelson et al. 2011
CORBIDI5840	*Rhinella margaritifera*	Peru	Curupa	KR012522	KR012612	KR012594	KR012564	This study
USNM268828	*Rhinella margaritifera*	Peru	Madre de Dios	-	DQ158490	DQ158490	-	Pramuk 2006
KU215145	Rhinella cf. margaritifera	Peru	Madre de Dios	-	DQ158491	DQ158491	-	Pramuk 2006
ZUEC-DCC3393	Rhinella cf. margaritifera	Brazil	Rio de Janeiro, Santo Aleixo	-	-	AY680262	-	Pauly et al. 2004
QCAZ17775	*Rhinella margaritifera*	Ecuador	244 km of Indanza	KR012529	KR012621	KR012581	KR012551	This study
QCAZ17989	*Rhinella margaritifera*	Ecuador	Estación Biológica JatunSacha	KR012530	KR012622	-	KR012565	This study
QCAZ17990	*Rhinella margaritifera*	Ecuador	Estación Biológica JatunSacha	KR012531	KR012623	KR012593	KR012557	This study
QCAZ17991	*Rhinella margaritifera*	Ecuador	Estación Biológica JatunSacha	KR012532	KR012614	-	KR012543	This study
QCAZ23632	*Rhinella margaritifera*	Ecuador	7Km North of Cosanga	KR012535	KR012627	KR012583	KR012542	This study
QCAZ23917	*Rhinella margaritifera*	Ecuador	Gualaquiza-El Ideal	KR012512	KR012628	KR012591	KR012547	This study
QCAZ10601	*Rhinella margaritifera*	Ecuador	Parque Nacional Yasuní	-	DQ15870	DQ15870	-	Pramuk 2006
QCAZ18241	*Rhinella margaritifera*	Ecuador	Shaime	KR012533	KR012625	KR012585	KR012553	This study
10226MSH	*Rhinella margaritifera*	Brazil	Amazonas, Anavilhanas	JN692056	JN691364	JN690757	-	Fouquet et al. 2012b
10339MSH	*Rhinella margaritifera*	Brazil	Amazonas, Anavilhanas	JN692057	JN601365	JN69058	-	Fouquet et al. 2012b
QCAZ42269	*Rhinella margaritifera*	Ecuador	Reserva Yachana	KR012541	KR012642	KR012584	KR012563	This study
111af	*Rhinella martyi*	French Guyana	Brownsberg	JN692045	EF364303	EF364277	-	Fouquet et al. 2007b
156mc	*Rhinella martyi*	French Guyana	Trijonction	EF364337	EF364303	EF364277	-	Fouquet et al. 2007b
LAJ210	*Rhinella ocellata*	Brazil	Tocantins, Lajeado	-	JN867572	JN867546	-	Fouquet et al. 2012a
MZUSP103261	*Rhinella ocellata*	Brazil	Tocantins, Peixe	-	DQ158479	DQ158479	-	Pramuk 2006
SMF88237	Rhinella cf. paraguayensis	Bolivia	-	-	JF790186	-	-	Jansen et al. 2011
MNKA9691	Rhinella cf. paraguayensis	Bolivia	-	-	JF790185	-	-	Jansen et al. 2011
ESTR00173	*Rhinella* sp.	Brazil	Amazonas, Carolina	-	JN867574	JN867548	-	Fouquet et al. 2012a
AF7275337	*Rhinella* sp.	Brazil	Mato Grosso, APM Manso	-	JN867575	JN867549	-	Fouquet et al. 2012a
**Outgroup**								
QCAZ50698	*Rhinella marina*	Ecuador	Puerto Cayo	KR012508	KR012643	KR012579	KR012545	This study
QCAZ50702	*Rhinella marina*	Ecuador	San Andrés de Rocafuerte	KR012509	KR012644	KR012580	KR012546	This study
QCAZ18203	*Rhinella festae*	Ecuador	Estación Biológica Jatun Sacha	KR012510	KR012624	KR012609	KR012574	This study
KU217501	*Rhinella festae*	Ecuador	Pastaza	-	DQ158423	DQ158423	-	Pramuk 2006
MTD43789	*Rhinella chavin*	Peru	Palma Pampa	-	DQ158441	DQ158441	-	Pramuk 2006
UTA53310	*Rhinella nesiotes*	Bolivia	La Paz	-	DQ158478	DQ158478	-	Pramuk 2006

## Results

### Phylogenetic analyses

The complete matrix contained up to four genes and 3045 bp for 92 samples. For the complete data set, PartitionFinder chose seven partitions as the best strategy (best model in parenthesis): 12S (GTR + I + G), 16S (GTR + I + G), COI 1^st^ position (TIMef + G), COI 2^nd^ position (TVM + I + G), COI 3^rd^ position (TrN + G), Tyr 1^st^ and 2^nd^ position (TrN + G), Tyr, 3^rd^ position (TrN + I + G). For the mitochondrial analyses, the same five partitions were chosen, one for each ribosomal RNA gene and each codon position in COI. For the nuclear analysis, two partitions were chosen: Tyr, 1^st^ and 2^nd^ position and Tyr, 3^rd^ position.

**Figure 3.**
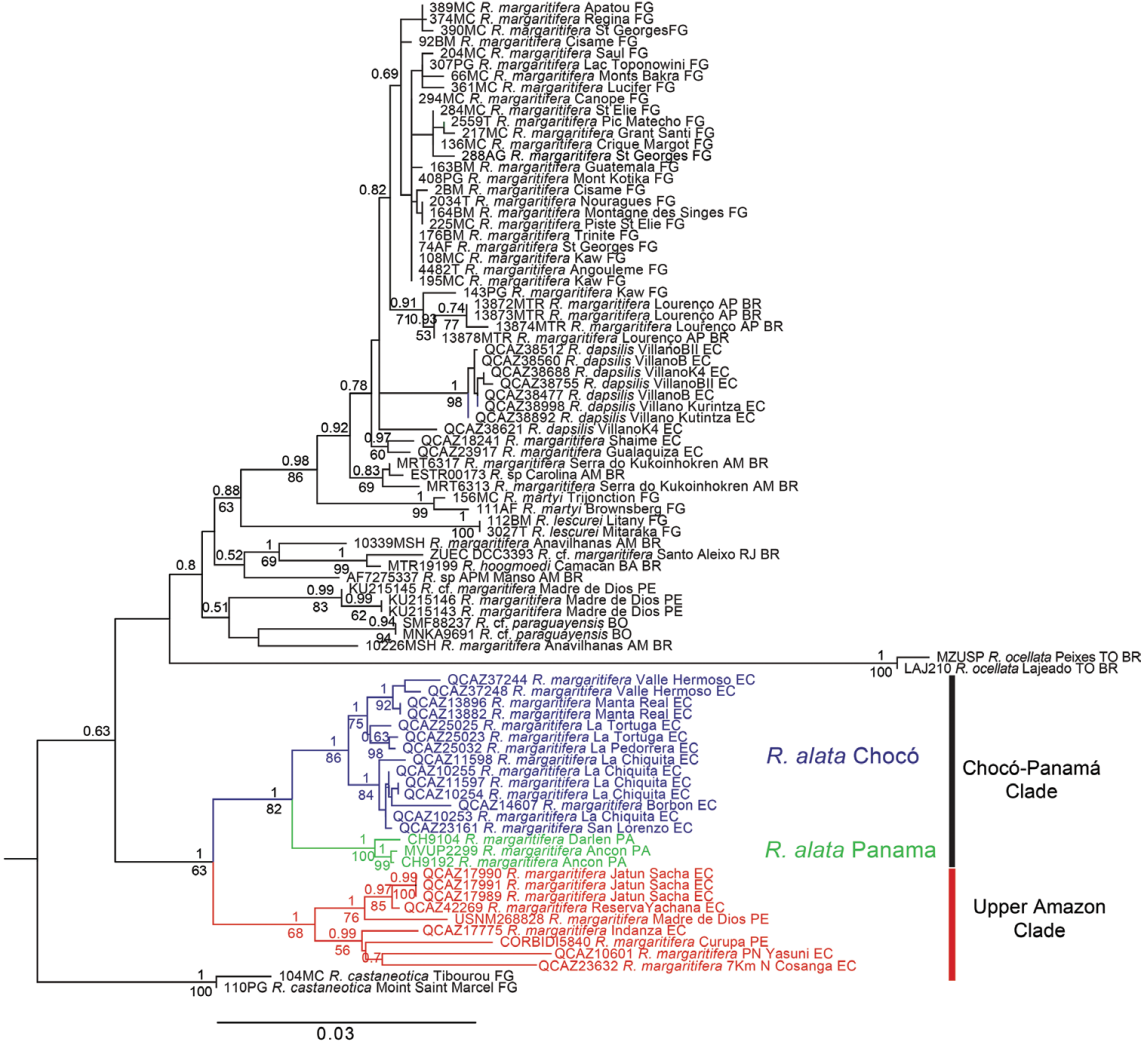
Maximum Likelihood phylogram depicting relationships within the *Rhinella margaritifera* species group. The phylogram was derived from the analysis of 3045 bp of mitochondrial (*12S*, *16S*, *COI*) and nuclear (*Tyr*) genes. Numeric codes on terminals are individual collection numbers (associated data listed in Table 1). Posterior probabilities (above) and bootstrap values (below) are shown on branches except when they are < 0.50 and 50%, respectively. Abbreviations are: EC = Ecuador, FG = French Guyana, BR = Brazil, BO = Bolivia, PE = Peru, PA = Panama. Outgroups are not shown.

The tree topologies for the Maximum likelihood and Bayesian phylogenies were similar except for weakly supported nodes (posterior probability < 0.95 and bootstrap < 75). The Maximum Likelihood tree (Fig. 3) shows a basal divergence of *Rhinella castaneotica*, which is sister to two clades containing the remaining species of the *Rhinella margaritifera* species group. One clade is strongly supported in the Bayesian consensus (posterior probability = 1) although it has low bootstrap support (= 63). It contains three groups: Panama (posterior probability = 1.0, bootstrap = 100), Chocó (posterior probability = 1.0, bootstrap = 86) and upper Amazon (posterior probability = 1.0, bootstrap = 68). Chocó and Panama form clade sister to the upper Amazon clade. Both clades, which are on opposite sides of the Andes, are separated by pairwise genetic distances (uncorrected *p* for the mitochondrial gene *16S*) ranging from 3.01 to 5.5% (average = 4.28, SD = 0.56). The genetic distances and the morphological differences (see next section) between the Chocó-Panama clade and the upper Amazon clade suggest that they are separate species. The *16S* genetic distances between the Chocó and Panama clades range from 1.26 to 1.99% (average = 1.63, SD = 0.19). The relatively low genetic distances and the lack of morphological differences between their populations (see next section) indicate that they are conspecific. The Chocó populations further segregate latitudinally in two well-supported clades. One includes the populations in northern Ecuador (e.g. Reserva La Chiquita and Borbón) while the other includes central and southern populations (e.g. Manta Real and Valle Hermoso, Fig. 3).

The sister clade to Chocó-Panama + Upper Amazon has weak support and includes other members of the *Rhinella margaritifera* group (*Rhinella dapsilis*, *Rhinella hoogmoedi*, *Rhinella lescurei*, *Rhinella martyi*, *Rhinella ocellata*, *Rhinella paraguayensis* and “*Rhinella margaritifera*”) from the Guiana region and Amazonian Brazil, Ecuador and Peru. Relationships among them are weakly supported on most branches.

**Figure 4.**
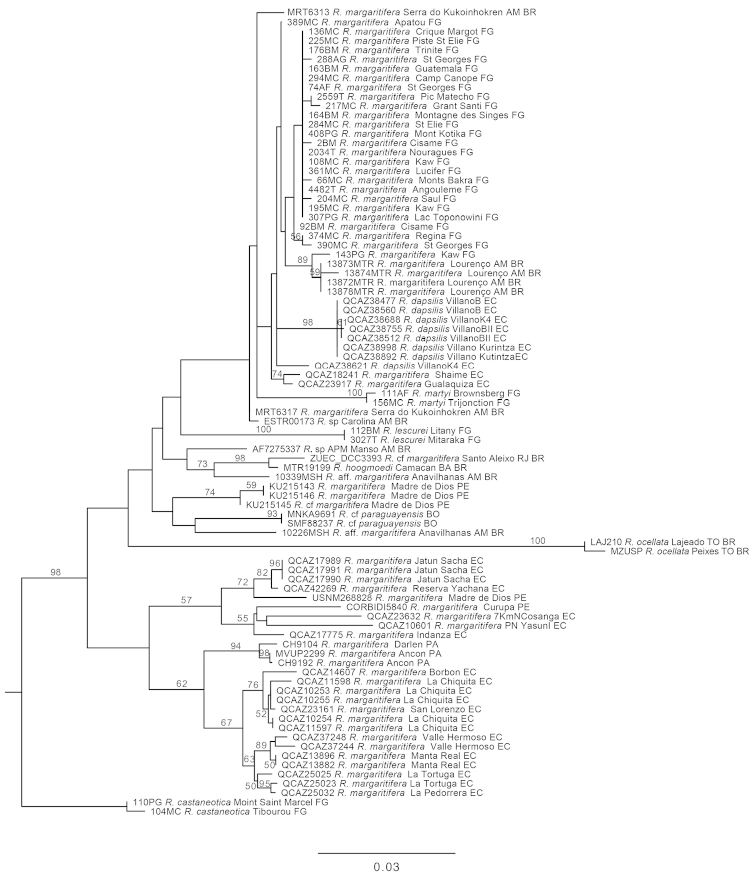
Maximum Likelihood phylogram depicting relationships within the *Rhinella margaritifera* species group. The phylogram was derived from the analysis of 2495 bp of mitochondrial gene fragments (*12S*, *16S*, *COI*). Numeric codes on terminals are individual collection numbers (associated data listed in Table 1). Bootstrap values appear above branches. The branches without numbers have bootstrap values < 50%. Abbreviations: EC = Ecuador, FG = French Guyana, BR = Brazil, BO = Bolivia, PE = Peru, PA = Panama. Outgroups are not shown.

The Maximum Likelihood tree based on mitochondrial genes (Fig. 4) has similar topology to the Maximum Likelihood tree derived from the analysis of the complete data set (Fig. 3). The Bayesian consensus tree, derived from the Tyrosinase gene, has definitely lower resolution (Appendix 2).

### Morphological analyses

**Figure 5.**
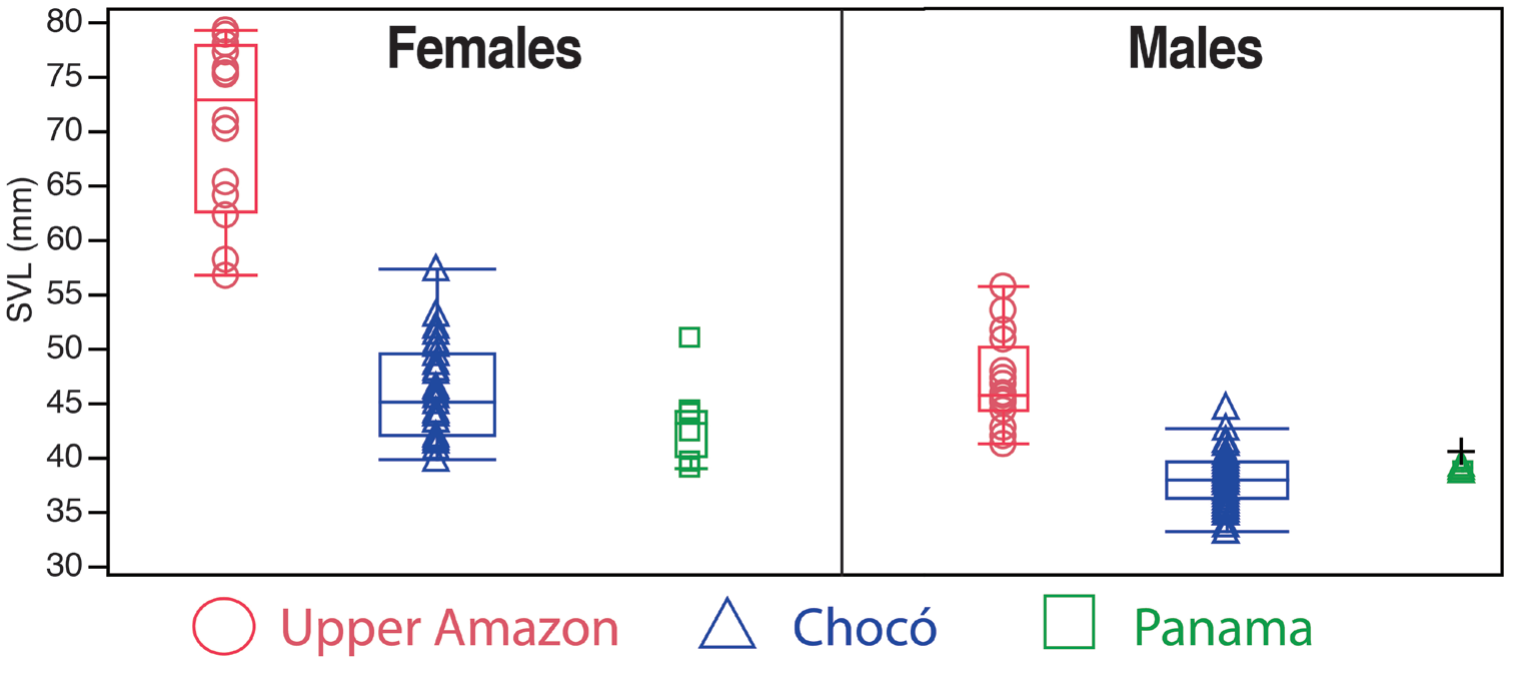
Box and whisker plots showing snout-vent length variation in adult *Rhinella margaritifera* (upper Amazon) and *Rhinella alata* (Chocó and Panama). The central bar indicates the median, the interquartile range is shown by the box length, and the range is shown by the short horizontal lines (whiskers). **SVL** = snout-vent length. The black cross is the holotype of *Rhinella alata*.

*Morphometric comparisons.* Morphometric data from adults are summarized in Table 2. In the examined series, Amazonian males and females were significant larger than their counterparts from Chocó (Fig. 5; males Student’s *t* = -10.32, DF = 57 *p* < 0.001; females *t* = -13.12, DF = 45, *p* < 0.001) and Panama (males *t* = -8.7, DF = 22, *p* < 0.001; females *t* = -4.43, DF = 20, *p* < 0.001). There are no significant differences in SVL between Chocoan and Panamanian populations (males *t* = 1.37, DF = 47, *p* = 0.91; females *t* = -1.58, DF = 37, *p* = 0.06).

**Table 2.** Descriptive statistics for morphometric measurements of adults from *Rhinella margaritifera* from Amazonian Ecuador and *Rhinella alata* from Chocó and Panama. Mean ± SD is given, with the range below. Abbreviations are: **SVL** = Snout-Vent Length; **TL** = Tibia Length; **FL** = Femur Length; **HL**= Head Length; **HW** = Head Width; **STCH** = Supratympanic Crest Height; **SOCH** = Supraorbital Crest Height; **NSD** = Nostril-Snout Distance; **IND** = Inter-Nostril Distance; **TD** = Tympanum Diameter; **FT** = Foot Length. All measurements are in mm.

	*Rhinella margaritifera*		*Rhinella alata*					
	Amazon		Chocó		Panamá		combined	
Morphometric measurements	Males (*n* = 16)	Females (*n* = 16)	Males (*n* = 43)	Females (*n* = 31)	Males (*n* = 6)	Females (*n* = 8)	Males (*n* = 49)	Females (*n* = 39)
**SVL**	45.6 ± 4.11 (54.36–39.88)	68.90 ± 8.26 (77.97–55.42)	36.66 ± 2.42 (43.25 –31.84)	44.82 ± 4.42 (56.19–38.55)	38.03 ± 0.59 (39.20–37.54)	42.38 ± 3.82 (49.69–37.78)	36.83 ± 2.31 (43.25–31.84)	44.27 ± 4.37 (56.19–37.78)
**TL**	18.73 ± 1.97 (23.13–15.14)	29.36 ± 2.97 (34.26–24.01)	15.98 ± 1.14 (18.72–13.69)	18.26 ± 1.24 (20.73–16.22)	15.86 ± 1.16 (18.12–15.09)	17.79 ± 0.75 (18.99–16.41)	15.97 ± 1.13 (18.72– 13.69)	18.17 ± 1.16 (20.73–16.22)
**FL**	19.67 ± 1.97 (23.84–16.15)	29.33 ± 3.67 (35.34–22.75)	15.69 ± 1.34 (19.28–13.09)	18.16 ± 1.72 (22.04–15.18)	16.39 ± 0.37 (17.01 –16.03)	17.46 ± 0.67 (18.72–16.72)	15.77 ± 1.27 (19.28–13.09)	18.02 ± 1.58 (22.04–15.18)
**HW**	16.9 ± 1.59 (19.93–14.77)	25.88 ± 2.73 (30.69–21.01)	12.57 ± 0.95 (15.14–10.31)	15.10 ± 1.6 (18.94–12.49)	12.98 ± 0.17 (13.3–12.8)	14.90 ± 1.12 (17.23–13.79)	12.63 ± 0.91 (15.14–10.31)	15.06 ± 1.50 (18.94–12.49)
**HL**	14.6 ± 1.28 (17.44–13.27)	22.27 ± 2.71 26.51–17.94)	11.61 ± 0.8 (13.88–10.29)	13.67 ± 1.19 (16.84–11.85)	11.85 ± 0.21 (12.2–11.54)	13.18 ± 1.12 (15.45–11.77)	11.64 ± 0.76 (13.88–10.29)	13.57 ± 1.17 (16.84–11.77)
**SOCH**	9.46 ± 0.86 (11.13–8.19)	15.43 ± 2.02 (18.33–12.06)	7.71 ± 0.59 (8.87–6.45)	9.28 ± 0.86 (11.40–7.77)	8.39 ± 0.21 (8.67–8.2)	9.13 ± 0.49 (9.87–8.53)	7.79 ± 0.59 (8.87–6.45)	9.25 ± 0.79 (11.4–7.77)
**STCH**	8.78 ± 1.55 (12.27–6.78)	17.73 ± 3.26 (22.7–12.35)	6.27 ± 0.54 (7.97–5.36)	7.96 ± 0.68 (9.71–6.63)	6.59 ± 0.31 (6.99–6.31)	7.38 ± 0.39 (8.01–6.99)	6.31 ± 0.52 (7.97–5.36)	7.84 ± 0.67 (9.71–6.63)
**NSD**	2.08 ± 0.44 (2.64–1.41)	2.45 ± 0.42 (3.37–1.79)	1.63 ± 0.29 (2.23–1.05)	1.70 ± 0.21 (2.08–1.25)	1.47 ± 0.17 (1.60–1.18)	1.66 ± 0.16 (1.89–1.35)	1.61 ± 0.28 (2.23–1.05)	1.69 ± 0.20 (2.08–1.25)
**IND**	3.35 ± 0.35 (3.89–2.70)	3.12 ± 0.37 (3.73–2.59)	2.50 ± 0.33 (3.23–1.86)	2.80 ± 0.43 (3.98–2.16)	2.41 ± 0.11 (2.59–2.31)	2.42 ± 0.23 (2.63–2.08)	2.48 ± 0.32 (3.23–1.86)	2.72 ± 0.43 (3.98–2.08)
**TD**	3.48 ± 0.24 (3.93–3.18)	4.14 ± 0.21 (4.48–3.65)	3.34 ± 0.47 (4.03–1.95)	3.46 ± 0.59 (4.45–2.5)	3.38 ± 0.20 (3.60–3.13)	3.79 ± 0.25 (4.05–3.31)	3.33 ± 0.45 (4.03–1.95)	3.52 ± 0.55 (4.45–2.5)
**FT**	16.87 ± 2.145 (21.85–13.76)	24.87 ± 3.64 (28.86 –19.13)	13.46 ± 1.12 (15.88–11.43)	15.33 ± 1.52 (19.40–13.15)	13.72 ± 0.68 (14.70–12.82)	14.96 ± 0.76 (16.54–14.39)	13.48 ± 1.07 (15.88–11.43)	15.25 ± 1.39 (19.4–13.15)

**Figure 6.**
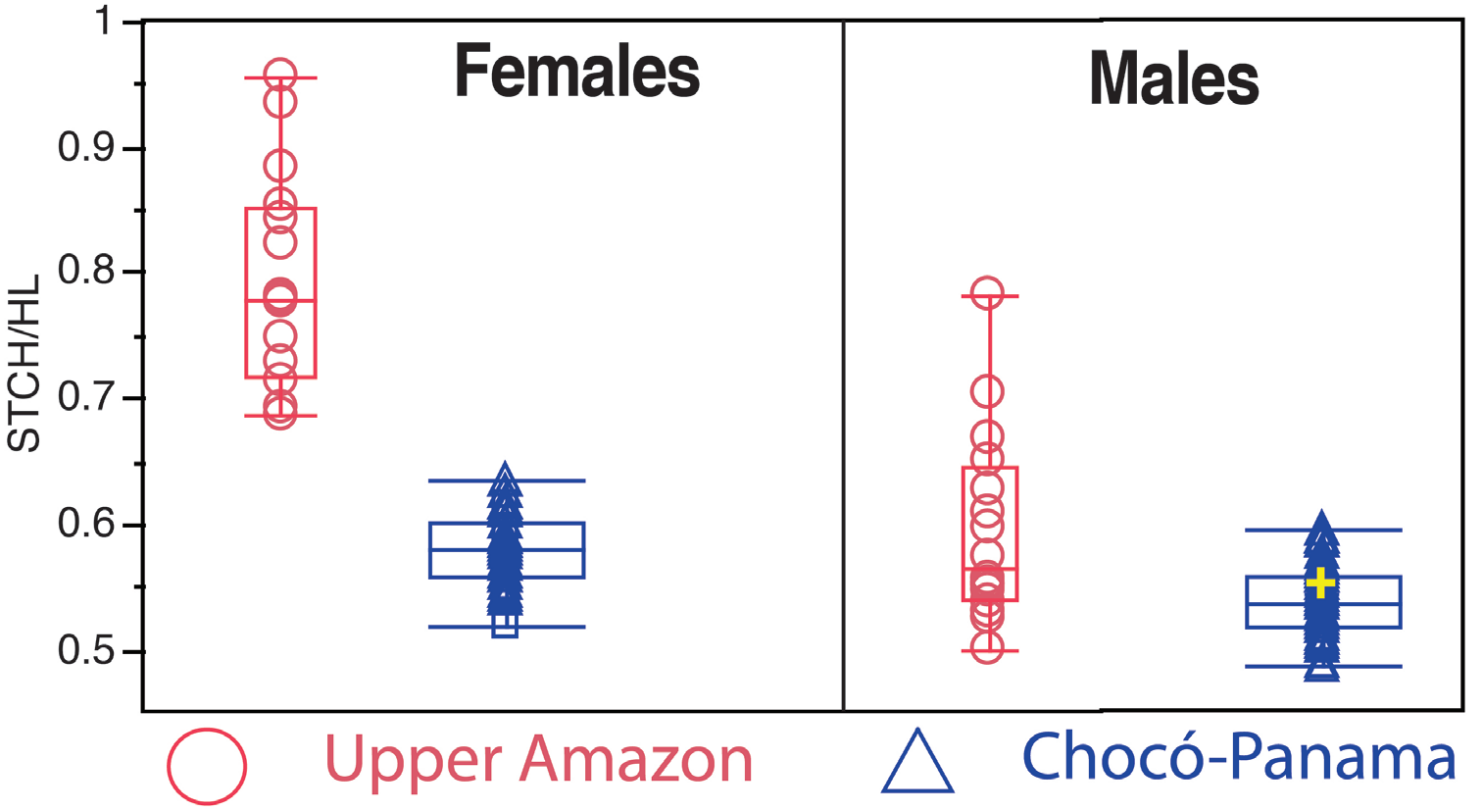
Box and whisker plots showing relative size of supratympanic crests for adult *Rhinella margaritifera* (upper Amazon) and *Rhinella alata* (Chocó-Panama). The central bar indicates the median, the interquartile range is shown by the box length, and the range is shown by the short horizontal lines (whiskers). **STCH** = supratympanic crest height, **HL** = head length. The yellow cross is the holotype of *Rhinella alata*.

Significant differences were observed in relative crest size between the Chocó-Panama and upper Amazon clades (Fig. 6). In the former, female supratympanic crest height had a range between 51.6 to 63.5% of head length (n = 39); in the later, range was 68.6 to 95.5% (n = 16). Ranges did not overlap and differences were significant (Wilcoxon’s *Z* = –5.77, *p* < 0.001). Male supratympanic crest height had a range between 49.3 to 59.8% of head length in Chocó-Panama (n = 49); in upper Amazon, range was 50.6 to 78.4% of head length (n = 16). Ranges overlapped but differences were significant (Wilcoxon’s Z = 3.11, *p* = 0.0018).

**Figure 7.**
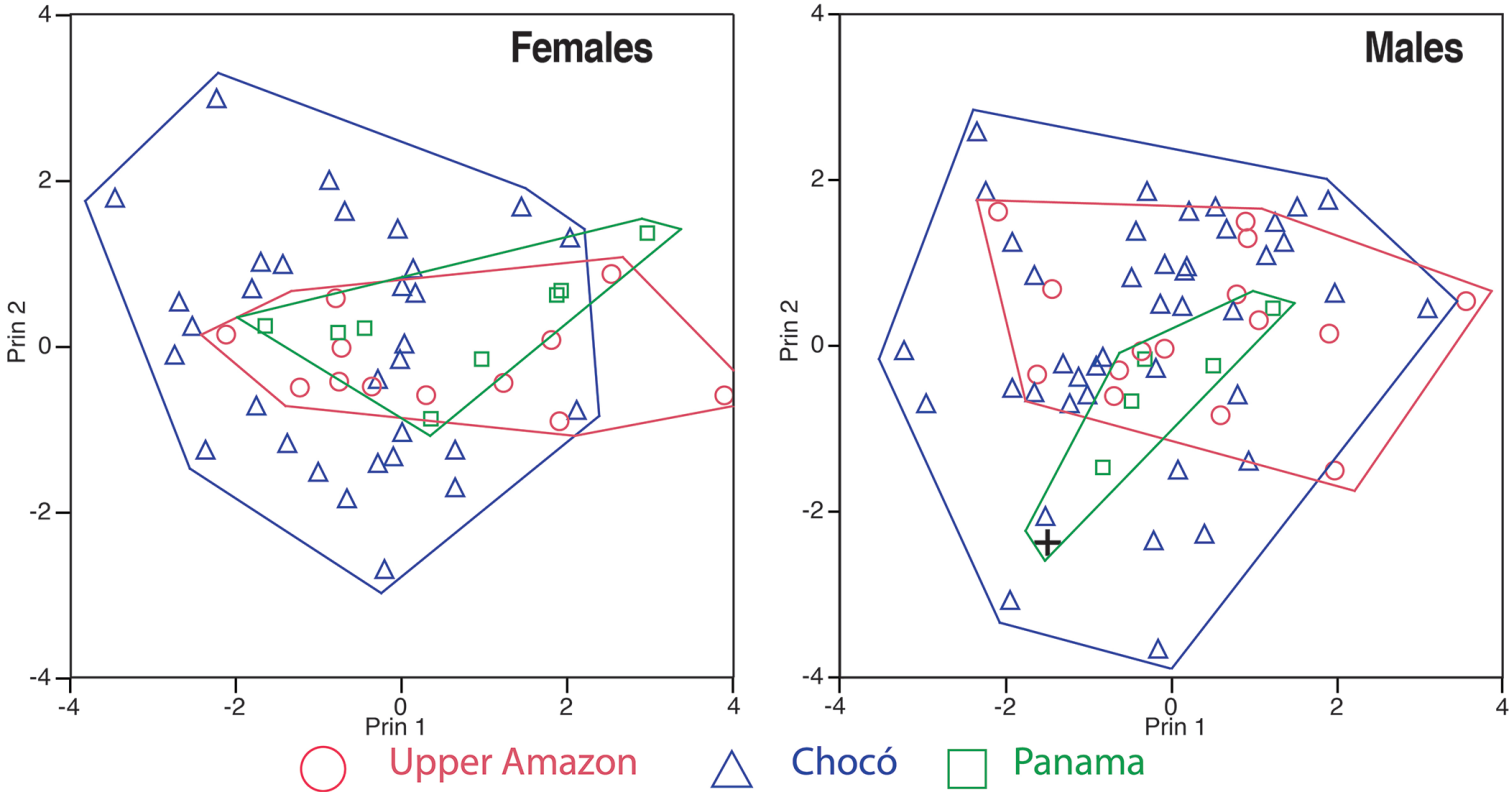
Principal components extracted from the analysis of ten size-corrected morphological variables of adult *Rhinella margaritifera* (upper Amazon) and *Rhinella alata* (Chocó and Panama). The black cross is the holotype of *Rhinella alata*. See Table 3 for character loadings on each component.

Three components with eigenvalues > 1.0 were extracted from the PCA for females (Table 3). The three components accounted for 67.3% of the total variation. The highest loadings of the PCA for females were supratympanic and supraorbital crest height, and tibia length for PC I, inter-nostril distance and tympanum diameter for PC II, and nostril-snout distance and inter-nostril distance for PC III. Three components with eigenvalues > 1.0 were extracted from the PCA in males (Table 3). The three components accounted for 63.3% of the total variation. The highest loadings for the PCA for males were head length and head width for PC I, inter-nostril distance and tympanum diameter for PC II, and tibia length and foot length PC III. The morphometric space of the Chocoan, upper Amazon, and Panamanian populations broadly overlaps in both males and females (Fig. 7).

**Table 3.** Character loadings and eigenvalues for Principal Components (PC) Analysis. The analysis was based on ten size-corrected morphometric variables measured in Amazonian, Chocoan and Panamanian populations of the *Rhinella margaritifera* species group. Abbreviations are: **TL** = Tibia Length; **FL** = Femur Length; **HL** = Head Length; **HW** = Head Width; **STCH** = Supratympanic Crest Height; **SOCH** = Supraorbital Crest Height; **NSD** = Nostril-Snout Distance; **IND** = Inter-Nostril Distance; **TD** = Tympanum Diameter; **FT** = Foot Length. Bold figures indicate highest loadings.

Variable	PCA Females			PCA Males		
	PC I	PC II	PC III	PC I	PC II	PC III
FL	0.330	0.165	0.167	0.272	0.159	0.322
FT	0.334	0.214	0.418	0.061	-0.038	**0.661**
HL	0.350	-0.065	0.153	**0.448**	-0.268	-0.078
HW	0.343	0.132	-0.288	**0.446**	-0.222	-0.045
IND	-0.203	**0.381**	**0.512**	0.280	**0.502**	-0.142
NSD	0.217	0.155	-**0.580**	0.262	0.386	-0.186
SOCH	**0.368**	-0.067	0.190	0.423	-0.071	-0.082
STCH	**0.411**	-0.154	-0.039	0.409	-0.290	-0.045
TD	0.071	**0.817**	-0.159	0.099	**0.557**	-0.128
TL	**0.368**	-0.200	0.232	0.134	0.228	**0.610**
Eigenvalue	4.411	1.192	1.128	2.800	1.947	1.585
Cumulative variance (%)	44.11	56.03	67.31	28.00	47.47	63.32

In the DFA classification for females, 51 out of 55 females were assigned correctly to their geographic region. The four misclassified females from Ecuadorian Chocó were assigned to Panamanian populations. All specimens from the upper Amazon were correctly classified. In the DFA for males, 56 out of 65 males were correctly classified. The eight misclassified males from Ecuadorian Chocó were assigned to Panamanian populations and only one from upper Amazon to Panamanian populations. All males and females from Panama were correctly classified. The DFA analyses indicate that populations from the Ecuadorian Chocó are morphometrically very similar with those from Panama, both groups being markedly different from *Rhinella margaritifera* from the upper Amazon.

Finally, evidence of sexual dimorphism was found in relative crest size: females have larger cephalic crests than males (Fig. 6). The ratio supratympanic crest height/head length (STCH/HL) was significantly different between males and females in the Chocó-Panama clade (Wilcoxon’s *Z* = 5.15, *p* < 0.001) and the upper Amazon clade (Wilcoxon’s *Z* = -4.35, *p* < 0.001).

### Qualitative morphological characters

**Figure 8.**
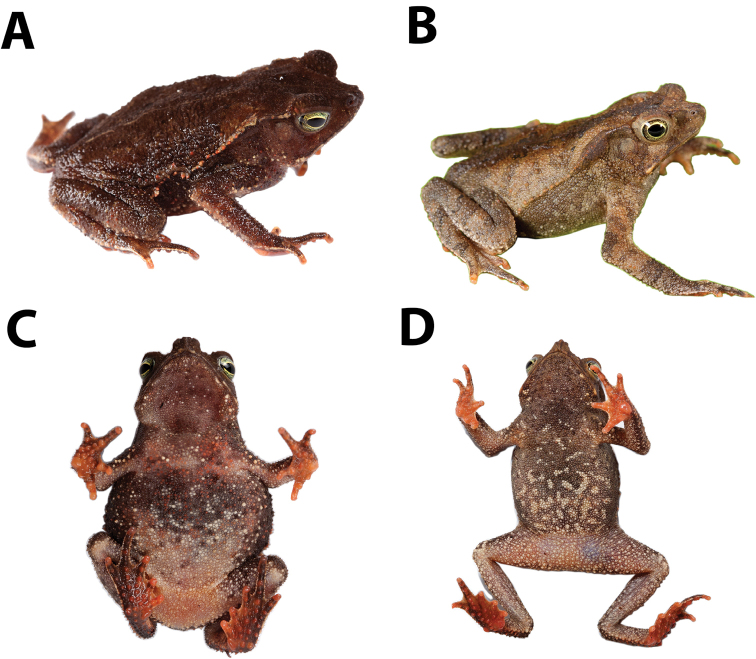
Dorsolateral and ventral views of *Rhinella alata* from the Chocó region. **A** and **C** QCAZ 50568 (SVL 40.37 mm), adult female, La Concordia, Santo Domingo Province, Ecuador **B** and **D** QCAZ 37248 (SVL 40.23 mm), adult male, Valle Hermoso, El Oro Province, Ecuador. Not shown at the same scale. Photos by S.R. Ron.

**Figure 9.**
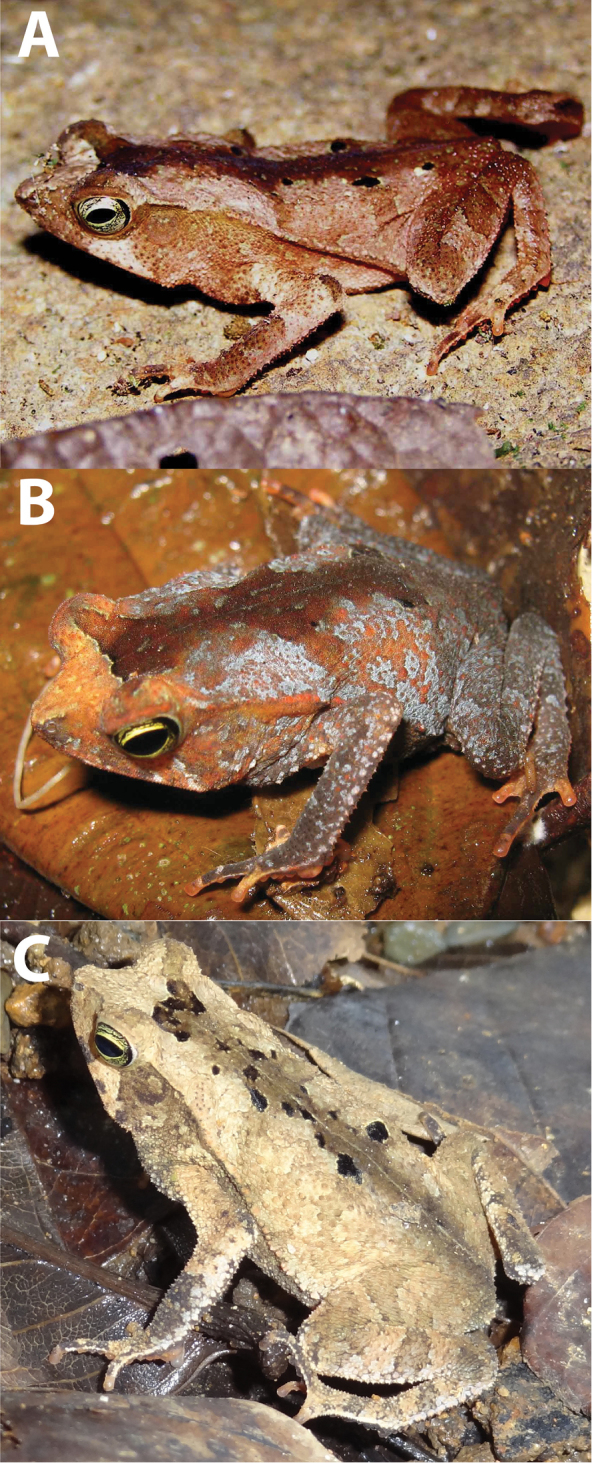
Dorsolateral views of *Rhinella alata*. **A** Cerro Azul, Parque Nacional Chagres, Panama Province, Panama. Photo by Ángel Sosa **B** Cerro Bruja, Parque Nacional Portobelo, Colón Province, Panama. Photo by Ángel Sosa **C** Gamboa, Colón Province, Panama. Photo by Roberto Ibáñez.

**Figure 10.**
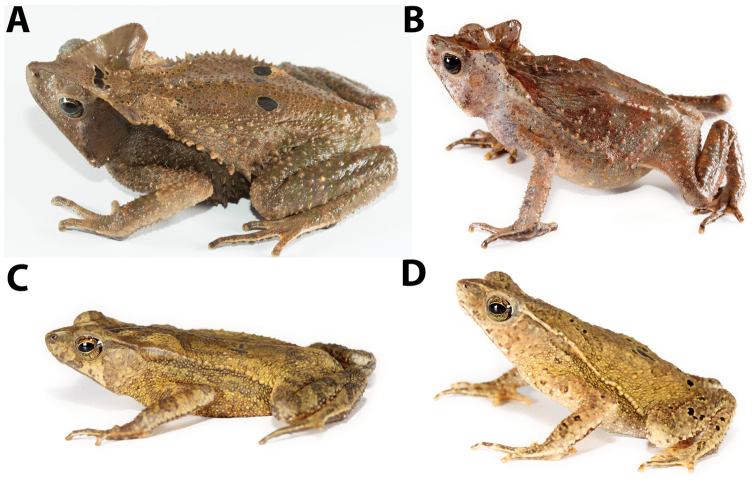
Dorsolateral views of *Rhinella margaritifera* from the Ecuadorian Amazon. Females: **A** QCAZ 55930 (SVL 80.15 mm) **B** QCAZ 55914 (SVL 72.49 mm), Lorocachi, Pastaza Province, Ecuador; males: **C** QCAZ 52343 (SVL 37.59 mm) **D** QCAZ 52344 (SVL 36.66 mm), Cascada San Rafael, Sucumbíos Province, Ecuador. Photos by S.R. Ron. Not shown at the same scale.

The upper Amazon clade differs from the Chocó-Panama clade in having protruding vertebral apophyses in the dorsum and bony knobs at angle of jaws (both absent in the Chocó-Panama clade; Figs 8–10). The Chocó-Panama clade differs from other species of the *Rhinella margaritifera* group by a combination of an an absence of vertebral apophyses, an absence of bony knob at angle of jaws, low cranial crests, and the tympanum rounded or ovoid (see *Systematic account* section). A large number of specimens were examined (see *Populations sampling* section) and all conform to this characterization. Thus, it seems unlikely that there are additional species of the group in the Chocoan and Panamanian regions.

**Figure 11.**
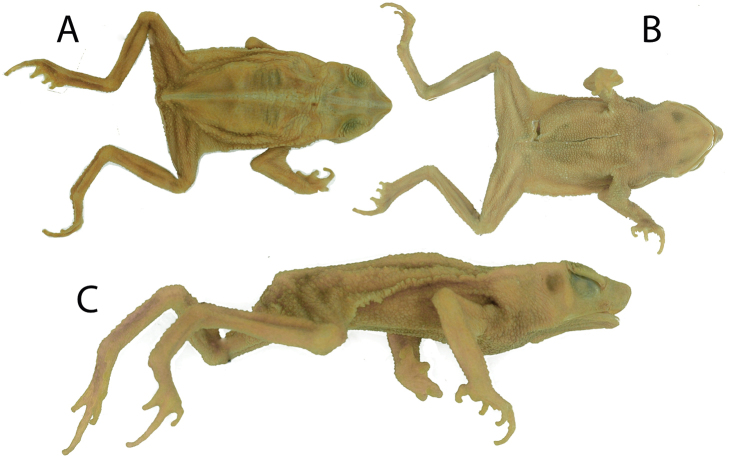
Dorsal (**A**), ventral (**B**), and lateral (**C**) views of the holotype of *Rhinella alata*. MNHN 84285, adult male, SVL = 39.2 mm.

The holotype of *Rhinella alata* (Thominot, 1884) (Fig. 11) is an adult male with an SVL of 39.2 mm. It has poorly developed supratympanic crests and lacks bony knobs at the angle of jaws. The vertebral apophyses are inconspicuous. These characters and the location of its type locality (within 6 km of one of our examined populations) lead us to conclude that it is conspecific with the Panamanian and Chocoan populations examined herein.

### Systematic account of *Rhinella alata*

#### Rhinella alata

Taxon classificationAnimaliaAnuraBufonidae

(Thominot, 1884)

Bufo alatus Thominot, 1884. Holotype: MNHN 84285, adult male from Obispo, Panama.

##### Diagnosis.

*Rhinella alata* is a small-sized (Table 2; Figs 8 and 9) species of *Rhinella* having the following combination of characters: (1) average SVL of females 44.25 mm (SD = 4.36, *n* = 39), males 36.83 mm (SD = 2.31, *n* = 49); (2) bony knob at angle of jaws absent, corner of mouth angular; (3) supraorbital crests low and thick, continuous with preorbital crests; usually with crenulate texture on vertical surfaces; (4) supratympanic crests concave and small; their posterior edge usually next to the anterior border of parotoid glands; (5) *canthus rostralis* present but inconspicuous, sometimes continuous with preorbital crests; (6) parietal crests usually present, ill-defined; (7) heel reaching posterior margin of eye when hindlimbs adpressed; (8) vertebral apophyses no protruding; (9) snout subacuminate in dorsal view, from rounded to protruding in profile; (10) skin on dorsum bearing a mixture of warts, pustules, and minute tubercles; (11) mid-dorsal line from snout to vent often present; (12) spiculate tubercles on external border of shank, evident especially on females; (13) dorsolateral row of sharply pointed, conical tubercles between posterior border of parotoid glands and groin; (14) tympanic membrane and tympanic annulus distinct; moderately large, ovoid to round; (15) parotoid glands small, elongated posteriorly; (16) upper eyelid warty; (17) tarsal fold absent; (18) digits slender and long, with small knobs at tip; lateral fringes present; finger lengths 3 > 4 > 2 > 1; toe lengths 4 > 5 > 3 > 2 > 1; (19) nuptial pads present.

*Rhinella alata* is most similar to *Rhinella acutirostris*. Both species differ from other members of the *Rhinella margaritifera* group by the absence of protruding vertebral apophyses, canthus rostralis not raised, snout projected, and low cranial crests. *Rhinella acutirostris* differs from *Rhinella alata* in having a bony knob at the angle of jaws (bony knob absent in *Rhinella alata* [Hoogmoed 1986, Lötters and Köhler 2000]). *Rhinella alata* differs from the holotype of *Rhinella proboscidea* (ZSM 1145/0) in having a less protruding snout and skin on dorsum bearing a mixture of warts, pustules, and minute tubercles (smooth skin in *Rhinella proboscidea*). *Rhinella dapsilis* is much larger than *Rhinella alata* (*Rhinella dapsilis* holotype SVL = 77 mm, adult male; Myers and Carvalho 1945) and has a fleshy proboscis in the snout (proboscis absent in *Rhinella alata*). *Rhinella alata* differs from *Rhinella yunga* in having tympanic membrane and annulus distinct (tympanic membrane and annulus absent in *Rhinella yunga*; Moravec et al. 2014). *Rhinella hoogmoedi*, *Rhinella magnussoni*, *Rhinella martyi*, *Rhinella paraguayensis*, *Rhinella scitula*, *Rhinella sclerocephala*, and *Rhinella stanlaii* have a bony knob at angle of jaws (Caramaschi and Pombal 2006, Lima et al. 2007, Fouquet et al. 2007a, Ávila et al. 2010, Caramaschi and Niemeyer 2003, Mijares-Arrutia and Arends-R 2001, Lötters and Köhler 2000; bony knob absent in *Rhinella alata*). *Rhinella alata* differs from *Rhinella castaneotica*, *Rhinella margaritifera* (sensu stricto) and *Rhinella roqueana*, by the absence of protruding vertebral apophyses (present in *Rhinella castaneotica* [Caldwell 1991], *Rhinella margaritifera* [Lavilla et al. 2013], and *Rhinella roqueana* [Melin 1941]).

*Rhinella alata* is most closely related to populations of *Rhinella margaritifera* from the upper Amazon basin in Ecuador and Peru. They can be easily distinguished by differences in body size (Fig. 5; see morphometric comparisons section) and relative size of cranial crests (Fig. 6).

##### Holotype.

The holotype is an adult male with SVL = 39.2 mm (Fig. 11). Descriptions of the holotype have been provided by Leavitt (1933) and Hoogmoed (1989). The bony knob at angle of jaws and vertebral apophyses are absent. The crests are low and thick. There is a dorsolateral row of conical tubercles from the posterior border of the parotoid gland to the groin. There is a clear mid-dorsal line from the snout to the vent. The tympanum is rounded.

##### Variation.

**Figure 12.**
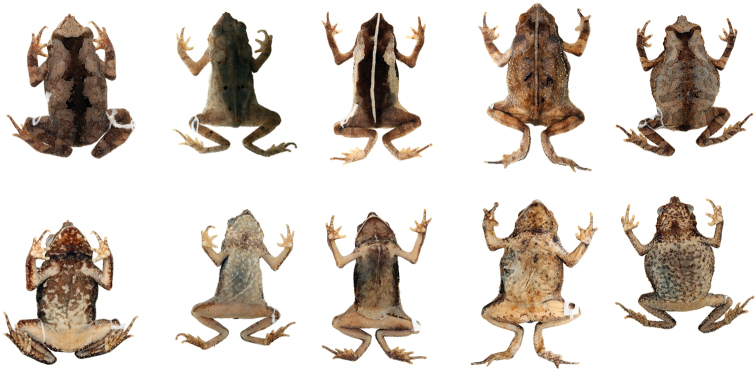
*Rhinella alata* from Ecuador showing variation in dorsal and ventral coloration of preserved specimens. Left to right, males: QCAZ 6733 (SVL 38.23 mm), QCAZ 10279 (SVL 35.08 mm); females, QCAZ 11598 (SVL 42.13 mm), QCAZ 14607 (SVL 50.95 mm), QCAZ 10439 (SVL 47.06 mm). See Appendix 1 for locality data. Not shown at the same scale.

**Figure 13.**
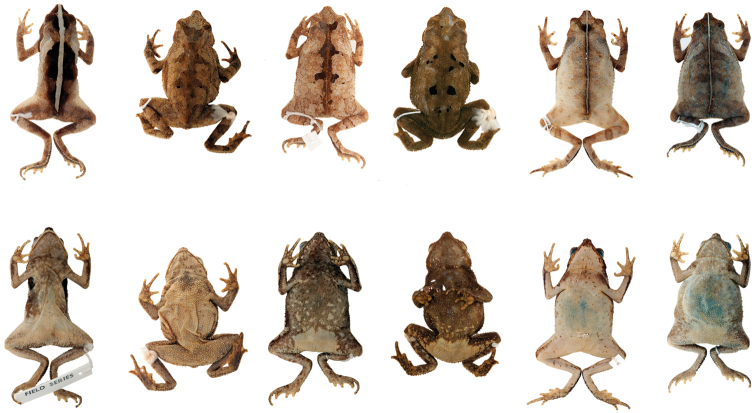
*Rhinella alata* from Panama showing variation in dorsal and ventral coloration of preserved specimens. Left to right, male: AMNH 89459 (SVL 37.54 mm); females, AMNH 88694 (SVL 41.21 mm), AMNH 55476 (SVL 41.19 mm), AMNH 104454 (SVL 49.69 mm), AMNH 88689 (SVL 42.75 mm), AMNH 20896 (SVL 42.98 mm). See Appendix 1 for locality data. Not shown at the same scale.

Variation in dorsal and ventral coloration of preserved specimens is shown in Figures 12 and 13. Background dorsal coloration varies from light gray (QCAZ 37244, AMNH 88689), light brown (QCAZ 14607, AMNH 104454) to dark gray (QCAZ 6733) or dark brown (QCAZ 11598, AMNH 52744), with irregular black and yellowish marks (QCAZ 4444, AMNH 88690). Some specimens have nearly uniform brown dorsum without marks (QCAZ 31603, 10296, AMNH 10296). A clear mid-dorsal line is often present (e.g. QCAZ 3502, QCAZ 12233).

Ventral surfaces of preserved specimens have a cream to yellowish-cream background color with irregular darker marks arranged in diverse patterns; marks can be light gray (QCAZ 6734, AMNH 88689), light brown (QCAZ 6732, AMNH 104454), dark gray (QCAZ 31606) or dark brown (QCAZ 6733, AMNH 89459), and vary from being restricted to the anterior half of the body (QCAZ 31604, AMNH 89459) to being present over the entire venter (QCAZ 4445, AMNH 88694). A longitudinal mid-ventral cream thin stripe can be present in the gular region (QCAZ 31602, 31606) or from the gular region to the mid-venter (QCAZ 6731, 11598).

Head shape in dorsal view varies from elongated (QCAZ 11598, AMNH 89459) to subtriangular (QCAZ 4447, AMNH 55475); in lateral view it varies from rounded (QCAZ 31605, AMNH 52749) to protruding (QCAZ 11393, AMNH 55475). Canthal region coloration varies from light gray or light brown to dark gray or dark brown. In some individuals the area below the eye and tympanum is yellowish cream (QCAZ 4447, AMNH 20896) or brown (QCAZ 31603, AMNH 88694) and differs from the color of the dorsum. Cloacal tubercles vary from yellowish cream (QCAZ 4441, AMNH 20896), to gray (QCAZ 31606) or brown (QCAZ 31602, AMNH 88695).

##### Color in life.

Based on digital photograph of an adult female QCAZ 50568 (Fig. 8). Dark brown dorsum with irregular light brown and yellowish marks; there is a clear mid-dorsal line. Dorsal surfaces of tights and shanks are dark brown with transversal brown bands. Dorsal surfaces of forelimbs are dark brown with irregular light brown marks. Dark brown tubercles are abundant on the dorsum. Ventral surfaces vary from light brown to dark brown, with some irregularly distributed white and orange spots. The fingertips and the subarticular tubercles on fingers and toes are red-orange. Canthal region and tympanum are dark brown; iris greenish yellow with black reticulation.

Based on a digital photography of an adult male QCAZ 37248 (Fig. 8). Light brown dorsum with black spots and light brown and light gray marks. Dorsal surfaces of tights, shanks and forelimbs are light brown with transversal dark brown bands. Brown tubercles are abundant on the dorsum. Ventral surfaces are dark brown with irregularly distributed yellowish marks; the posterior part of the venter is cream. The subarticular tubercles of palms, soles, and fingertips are red-orange. Canthal region and tympanum are dark brown; iris greenish yellow with black reticulation.

##### Distribution and ecology.

*Rhinella alata* has been recorded at 37 localities in the Ecuadorian Chocó (Cañar, Carchi, El Oro, Esmeraldas, Manabí, Pichincha, and Santo Domingo Provinces; Fig. 1), one locality in the Colombian Chocó (Barbacoas, Nariño; see *Taxonomic remarks*) and 35 localities in Panama (Comarca Guna Yala and Provinces Coclé, Colón, Darién and Panama; Fig. 2). It has a wide elevation range, from 19 to 1500 m above sea level.

The examined specimens from Chocoan populations contain 21 gravid females (average SVL = 45.37 mm, SD = 4.05 mm): QCAZ 4262, QCAZ 4441, QCAZ 4442, QCAZ 4443, QCAZ 7065, QCAZ 10296, QCAZ 11597, QCAZ 11598 collected in January; QCAZ 50568 collected in February; QCAZ 11392, QCAZ 31601, QCAZ 31603, QCAZ 31605 collected in April; QCAZ 25023 collected in June; QCAZ 10439 collected in August; QCAZ 14607 collected in November; QCAZ 10301 collected in December. This suggests year round reproductive activity with a peak between January and April, a period that corresponds to the rainy season in the Ecuadorian Chocó.

In Panamanian populations gravid females were found in January (AMNH 104454), September (AMNH 55461), November (AMNH 88689), and December (AMNH 53699). In central Panama, *Rhinella alata* breeds in ponds and pools along permanent streams or swamps. Reproduction is explosive and most takes place from the middle of the rainy season to early dry season (Wells 1979, Ibáñez et al. 1999). Choruses last less than 24 hours with males usually calling at night and oviposition occurring by day, especially in the early afternoon (Wells 1979). Otherwise, individuals are primarily diurnal, found active on the leaf litter of the forest floor during daytime, and often found asleep on leaves of low vegetation at night (Ibáñez et al. 1999). Diet is specialized on ants (Toft 1981).

Most of the Ecuadorian specimens are from Reserva Mayronga and Reserva Ecológica Cotacachi-Cayapas. They were found in the leaf litter of secondary forest and in agricultural lands. Some adults were observed at night within the forest in vegetation above the ground and some were found in amplexus (QCAZ 10271, QCAZ 10274, QCAZ 10275 in November 1996, and QCAZ 31604, QCAZ 31605 in February 1996). All the specimens collected in Reserva Ecológica Cotacachi-Cayapas were found in secondary forest. At some collecting sites, the forest has been cleared for cacao plantations (QCAZ specimen database).

According to the classification of Sierra et al. (1999) the vegetation types for Ecuadorian localities are: (1) Lowland Evergreen Forest of Coastal Range, characterized by abundant epiphytes, climbers and herbaceous plants, with a canopy of 30 m (e.g. Reserva La Chiquita, Durango); (2) Semideciduous Lowland Forest of Coastal Range, defined by the presence of broad canopy trees up to 20 m and curved shafts; the tree stratum is characterized by the presence of spiny, deciduous species with epiphytes while the forest floor has herbaceous plants (e.g. Bilsa, La Tortuga); (3) Evergreen Foothill Forest of Coastal Range, characterized by a canopy that can reach 30 m or more and trunks of trees covered with orchids, bromeliads, ferns and aroids (e.g. Manta Real, Alto Tambo); (4) Deciduous Lowland Forest of Costal Range, characterized by losing leaves during part of the year with a great varieties of cactus and thorny plants; the most conspicuous trees are the family Bombacaceae have curved trunks and broad crown. (e.g. El Progreso); (5) Semideciduos Foothill Forest of Coastal Range, characterized by having slightly dispersed vegetation, with trees over 20 m and dense herbaceous layers of ferns (e.g. Valle Hermoso).

The main vegetation types for Panamanian localities are (following Hogan 2010): (1) Isthmian-Atlantic Moist Forests, characterized by tall tropical evergreen forest with buttressed canopy trees reaching 40 m and with an extremely rich epiphyte flora (e.g. Cruces Trail, Punta Rincón); (2) Eastern Panamanian Montane Forest, at elevations from 500 to 1800 m above sea level, includes marshes, swamp forests, semi-deciduous tropical moist forests, premontane wet forest, cloud forests and elfin forests (e.g. Cana, Cerro Tacarcuna); (3) Chocó-Darién Moist Forests, at elevations between 0 and 1000 m above sea level, between the Pacific Ocean and the western range of the Andes (e.g. Dad Nakue Dubpir, Udirbi).

##### Taxonomic remarks.

Based on morphological characters, Vélez-Rodriguez (2004) ascribed four populations from Panama and Colombia to *Rhinella alata*: Isthmus of Panama (Panama; 15 males, 10 females); Parque Nacional Los Katíos (Colombia; 12 males, 15 females); Gorgona and Güape Island (Colombia; 7 males, 8 females); Municipio Restrepo (Colombia; 7 males, 8 females). Based on data from Vélez-Rodriguez (2004), these populations differ from the holotype of *Rhinella alata* and populations of *Rhinella alata* in Ecuador and Panama (in parentheses) in having: (1) a *canthus rostralis* protruding in females and ill-defined in males (inconspicuous in males and females), (2) parietal crests well defined in females, ill-defined in males (ill-defined in males and females), (3) vertebral apophyses slightly visible externally (absent). The differences suggest that those specimens are not *Rhinella alata* and may belong to a different species. Alternatively, differences between *Rhinella alata* described by Vélez-Rodriguez (2004) and our study could be an artifact resulting from the use of distinct terminology for similar character states.

In contrast, Mueses-Cisneros and Moreno-Quintero (2012) reported two species of the *Rhinella margaritifera* group form Barbacoas, Nariño, Colombia (*Rhinella* sp. 9 and *Rhinella* sp. 10). Two photographs of live individuals (pp. 45) show morphological features that fall within the observed variation of *Rhinella alata*. We tentatively assign them to *Rhinella alata* but direct specimen examination is required to confirm this identification.

## Discussion

The taxonomic status and phylogenetic position of populations traditionally ascribed to *Rhinella margaritifera* (= *Bufo typhonius*; e.g. Anderson 1945, Miyata 1982, Ortega-Andrade et al. 2010) from western Ecuador and Central America were reviewed. The examination of the holotype of *Rhinella alata* in combination with the morphological and genetic information from 72 populations from the Chocó region and Panama, indicate that those populations should be referred to *Rhinella alata*. The similarity between Chocoan and Panamanian populations was previously noted by Hoogmoed (1990).

### Systematics and morphology

Hoogmoed (1990), Lescure and Marty (2000) and Fouquet et al. (2007b) considered that *Rhinella margaritifera* from French Guyana, with hypertrophied crests, corresponds to *Rhinella margaritifera* sensu stricto. In a recent review, however, Lavilla et al. (2013) assigned a neotype with the type locality in “Humaitá, State of Amazonas, Brazil”. In our phylogeny (Fig. 3), the sister clade of *Rhinella ocellata* include the closest localities to the new type locality for *Rhinella margaritifera* and are likely to contain populations of *Rhinella margaritifera* sensu stricto. Our phylogeny and previous reviews (e.g. Fouquet et al. 2007b) indicate that species diversity in the *Rhinella margaritifera* group is greatly underestimated. In our phylogeny, two *Rhinella margaritifera* from the southern Amazon in Ecuador (QCAZ 18241 and QCAZ 23917) are more closely related to *Rhinella margaritifera* from French Guyana and *Rhinella dapsilis* than to other *Rhinella margaritifera* from Amazonian Ecuador. They probably represent an undescribed species, characterized by the presence of vertebral apophyses, bony knobs at the angle of jaws, and poorly developed crests. More studies are needed to define the status of these populations, as well as that of Rhinella cf. paraguayensis from Bolivian and Brazilian Amazon and Rhinella cf. hoogmoedi from Brazilian Atlantic Forest.

The identity of the upper Amazon clade (Ecuador-Peru) remains unresolved. It was not possible to ascribe it unequivocally to any described species of the *Rhinella margaritifera* species group and it is unlikely to be *Rhinella margaritifera* sensu stricto (as defined by Lavilla et al. 2013). Thus, these populations may belong to an undescribed species characterized by having prominent supratympanic crests, conspicuous vertebral apophyses in the dorsum and bony knobs at angle of jaws (Fig. 10). We refrain from describing this species until genetic samples of *Rhinella margaritifera* sensu stricto are available and a comprehensive review of the group is carried out. For now, we suggest that these populations are referred as *Rhinella margaritifera* sensu lato.

These results raise some rather interesting questions. For instance, the complete distribution range of *Rhinella alata* is yet to be determined. Extensive and explicit studies are necessary to reveal whether the species is continuously distributed from Ecuador to Panama or if it consists one, two (or more) disjoint population nuclei. This would be an indispensable step before planning further studies on the evolutionary history or conservation status of the species. Moreover, future studies including a larger number of samples, more representative of the geographic range of each species within the *Rhinella margaritifera* group, from Colombia, Venezuela and Suriname, will help to clarify their evolutionary identity. It will also be necessary to re-evaluate, using molecular, morphological, ecological, behavioral, and phylogenetic analyses, the taxonomic status of species that have been previously described only morphologically such as *Rhinella acutirostris*, *Rhinella magnussoni*, *Rhinella proboscidea*, *Rhinella roqueana*, *Rhinella sclerocephala*, *Rhinella scitula* and *Rhinella stanlaii*. Integrative approaches like the one we pursued in this study will help to disentangle the complex evolutionary history, systematics, and taxonomy of this species group.

### Biogeographic implications

Because all species in the *Rhinella margaritifera* species group are distributed in South America, it is reasonable to assume that the presence of *Rhinella alata* in Central America is the result of a single dispersal event from South America. The genetic distances between Chocoan and Panamanian populations are low (range 1.2–1.9%) and suggest that their divergence was recent and occurred after the closure of the Panamanian isthmus during the late Pliocene. Assuming a rate of evolution of the gene *16S* of 0.00249–0.00277 substitutions per site per lineage per Myr (Evans et al. 2004; Lemmon et al. 2007), the divergence between these populations occurred ~ 2.16 to 3.42 Myr ago (under the 0.00277 rate) or ~ 2.41 to 3.81 Myr ago (under the 0.00249 rate). Thus, it is likely that the divergence between Panama and Chocó took place after the completion of the Panamanian Isthmus (~ 3.5 Myr ago; Coates et al. 1992, Coates and Obando 1996). These estimates of time of divergence, however, should be considered with extreme caution because they assume a molecular clock at a rate estimated for species in different families. Further explicit studies will be necessary to estimate divergence times with more confidence.

*Rhinella alata* is sister to populations of *Rhinella margaritifera* from the Ecuadorian and Peruvian Amazon and the eastern Andean slopes, up to 2000 m of elevation, forming altogether a robust clade. The two lineages are highly divergent from each other (uncorrected *p* distances 3.0–5.5%, mitochondrial gene *16S*) and are morphologically distinctive. Therefore, both clades clearly represent separate species. Previously, *Rhinella margaritifera* was considered to occur on lowland rainforests east and west of the Andes of Ecuador. This distribution was atypical because out of 174 amphibian species inhabiting the Amazonian rainforests of Ecuador below 600 m of elevation, only three also occur in the rainforests of the Chocó region west of the Andes: *Hypsiboas boans*, *Rhinella marina* and *Trachycephalus typhonius* (Ron et al. 2014). Despite having similar environmental conditions and being geographically close (as low as 100 km of airline distance), rainforests on both sides of the Andes share few amphibian species, a result of the barrier effect of the Andes. Our results showing that *Rhinella margaritifera* only occurs on the eastern side demonstrate that their unusual distribution was an artifact of the incorrect delimitation of species boundaries. We suspect that the same problems could explain the disjunct distributions of *Rhinella marina*, *Trachycephalus typhonius* and *Hypsiboas boans*. Therefore, tropical rain forests of the Amazon and the Chocó may not share amphibian species.

## Supplementary Material

XML Treatment for Rhinella alata

## Figures and Tables

**Figure:**
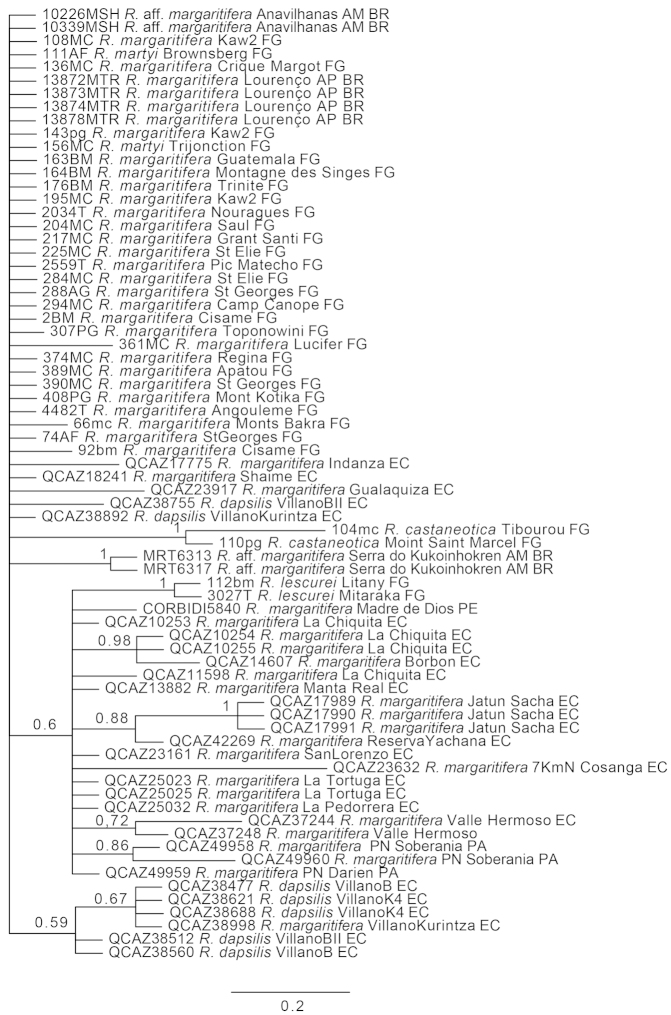
Bayesian consensus phylogram depicting relationships within the *Rhinella margaritifera* species group. The phylogram was derived from the analysis of 550 bp of nuclear gene Tyrosinase. Museum catalog numbers are shown in Table 1. Abbreviations are: EC = Ecuador, FG = French Guyana, BR = Brazil, BO = Bolivia, PE = Peru, PA = Panama. Outgroups are not shown.

## Appendix 1

Examined material. Numbers in bold indicate specimens analyzed genetically and morphometrically.

*Rhinella alata*.— ECUADOR: PROVINCIA CAÑAR: Manta Real, Río Patul (2.5679°S, 79.3666°W), 350–400 m (QCAZ 3437, 3551, 4757–758); Manta Real (2.5537°S, 79.3642°W), 500 m (QCAZ 12778–779). PROVINCIA CARCHI: Vía Zumba–El Chota, 1500 m (QCAZ 12233). PROVINCIA EL ORO: Valle Hermoso, Parroquia Bella María (3.5019°S, 79.8172°W), 379 m (**QCAZ 37244, 37248**); El Progreso, vía Pasaje–Pan de Azúcar (3.2883°S, 79.7581°W), 180 m (QCAZ 10366). PROVINCIA ESMERALDAS: Lagarto, Mayronga Reserve (1.042°S, 79.28°W), 100 m (4262–4264, 4441–4451, 4709–4717, 6637–6642); Reserva Ecológica Bilsa (0.6202°S, 79.931°W), 534 m (QCAZ 6731–6743); Corriente Grande, Río Cayapas (0.6895°S, 78.9589°W), 70 m (QCAZ 10271, 10274–281, 10289, 10290, 10292, 10295–299, 10299, 10301); Reserva Ecológica Cotacachi Cayapas, Charco Vicente (0.6962°S, 78.9109°W), 60 m (QCAZ 3338–3339, 11391–396); Pichiyacu, Comunidad Chachi, Río Cayapas (0.9081°S, 78.998°W), 260 m (QCAZ 31602–609); Reserva Ecológica Cotacachi–Cayapas o Playa de Oro (0.8285°S, 78.722°W), 179 m (QCAZ 49381–382, 49387, 49391); Las Golondrinas near Río Canandé (QCAZ 12651–652); Durango, Río San José (1.054°S, 78.625°W), 33 m (QCAZ 24968–978); Río Onzole (0.712°S, 79.092°W), 110 m (QCAZ 10440–443); Comunidad Loma Linda, Río Onzole (0.8754°S, 79.0511°W), 95 m (QCAZ 10439); La Concordia (0.0022°S, 79.4105°W), 144 m (QCAZ 50573, 50568); San Lorenzo, Protectora La Chiquita (1.2333°S, 78.76°W), 60 m (**QCAZ 10253, 10254**–255, **11597**, **11598**); San Lorenzo, La pera del Guarapo (1.2684°S, 78.8067°W), 253 m (**QCAZ 23161**); La Pedorrera (0.4667°S, 79.9833°W), 53 m **(QCAZ 25032)**; La Tortuga (0.591°S, 79.957°W), 86 m (**QCAZ 25023**); Borbón (1.0667°S, 79.05°W), 70 m (**QCAZ 14607**); Viche (0.6615°S, 79.5387°W), (QCAZ 4674); Durango (1.0427°S, 78.6245°W), (QCAZ 8549, 35250); 7 km western of Durango (1.0133°S, 78.6682°W) 220 m, (QCAZ 23164, 23623); Viruela, Rio Cayapas (1.1142°S, 78.9936°W), 45 m (QCAZ 10289); Al Tambo (0.9169°S, 79.5662°W) 253 m, (QCAZ 21138); El Milagro, La Mayronga (1.003°S, 79.326°W). PROVINCIA MANABÍ: El Carmen (0.274°S, 79.459°W). 300 m (QCAZ 7038–7039, 7065). PROVINCIA PICHINCHA: Reserva Forestal ENDESA (0.1667°S, 79,1667°W), 720 m (QCAZ 1659); Río Canoi (0.075°S, 79.051°W), 570 m (QCAZ 2745); 1 km E of Pedro Vicente Maldonado (0.0833°S, 79.039°W), 670 m, (QCAZ 2752); San Miguel de los Bancos (0.0166°S, 78.8833°W), (QCAZ 3813, 3815–818); San Miguel de los Bancos, Río Pitzará, 130 m (QCAZ 50846); km 9 San Miguel de los Bancos–Puerto Quito road (0.072°S, 78.9599°W), (QCAZ 5860); Puerto Quito, ENDESA (0.098°S, 79.117°W), (QCAZ 36827). PROVINCIA SANTO DOMINGO: Bosque Protector La Perla (0.057°S, 79.359°W), (QCAZ 3500–504); km 8 road to Santo Domingo (0.2005°S, 79.1924°W), 528 m (QCAZ 23621). PANAMA: COMARCA GUNA YALA: Dad Nakue Dubpir, Río Ogandí (9.2477°N, 78.1744°W), 150 m (CH 8842); Udirbi, Reserva Forestal (9.3167°N, 78.9833°W), 342 m (CH 1706); PROVINCIA COCLÉ: La Mina, Río Indio (8.9382°N, 80.1469°W), 48 m (CH 4922); near Río Tife cascade, Parque Nacional General de División Omar Torrijos Herrera (8.7065°N, 80.6352°W), 460 m (CH 0065); Obispo (9.1167°N, 79.6833°W) (MNHN 84285); Quebrada La Tiburcia, Cascajal (8.7158°N, 80.4605°W), 180 m (CH 5042); Quebrada La Varona, near Palmarazo (8.7342°N, 80.6565°W), 125 m (CH 5139). PROVINCIA COLÓN: Chitra, Santa Isabel (9.5186°N, 79.1534°W), 90 m (CH 7783); El Limón, Río Indio (8.9919°N, 80.1701°W), 19 m (CH 4967); Rinconcito, Punta Rincón (9.0135°N, 80.6884°W), 52 m (CH 1412); Río Caimito, Petaquilla (8.9706°N, 80.671°W), 54 m (CH 5476); Río Boquerón (9.3857°N, 79.4826°W), 150 m (AMNH 89459); Río Frijoles, Camino del Oleoducto, Parque Nacional Soberanía (9.1523°N, 79.7347°W), 67 m (CH 0307); road to Piña, after the represa Gatún (9.2603°N, 79.94°W), 34 m (CH 1679); Sta. Rosa and Guayabalito (9.1833°N, 79.65°W), 36 m (AMNH 55475); PROVINCIA DARIÉN: between Dos Bocas de Antaral and campsite on Serranía de Jingurudó (7.6564°N, 77.9986°W), <675 m (CH 4641); Cerro Tacarcuna, Río Pucuro (8.0011°N, 77.4852 °W), 640 m (AMNH 104454); Cana, trail to Boca de Cupé, Pinogana (7.7661°N, 77.6752°W), 518 m (CH 9104); Estación Pirre, Río Peresénico (8.0192°N, 77.7325°W), 90 m (CH 4057); Laguna Purriche (7.7222°N, 77.6555°W), 475 m (CH 6376); PROVINCIA PANAMA: Altos de Majé (AMNH 88689–8690, 88694); Barro Colorado (9.1636°N, 79.8378°W), 79 m (AMNH 20896, 5274, 55461–462); Parque Nacional Soberanía, Ancón (9.0764°N, 79.6594°W), 130 m (CH 9192); Chiva Chiva Road, Parque Nacional Camino de Cruces (9.0284°N, 79.5899°W), 41 m (CH 0491); Cruces trail (9.0453°N, 79.5892°W), 77 m (AMNH 55460); Finca Santa Bárbara, Nuevo Emperador, Arraiján (9.0011°N, 79.7235°W), 135 m (CH 1158); near Boquerón, Candelaria and Peluca (9.3671°N, 79.5546 °W) (AMNH 53699); near entrance to Chilibrillo Cave (9.1833°N, 79.6167°W) (AMNH 55476); Pacora (9.0833°N, 79.2833°W), 20 m (QCAZ 55481); Río Arraijancito (8.983°N, 79.6361°W), 110 m (CH 3980); Río Chico Masambí, Parque Nacional Soberanía, Ancón (9.0787°N, 79.6601°W), 135 m (MVUP 2299); Río Indio Arriba (8.6562°N, 80.1144°W), 645 m (CH 5005); San Juan de Pequení (9.3841°N, 79.5227°W), 100 m (CH 3702); stream near ACP Estación Río Chico (9.2636°N, 79.5097°W), 116 m (CH 6825); Tortí (8.9389°N, 78.4573°W), 95 m (MVUP 2256); Trinidad (8.7321°N, 79.9617°W), 420 m (CH 4313); Altos de Cerro Azul, Cerro Jefe (9.2284°N, 79.4046°W), 800 m (CH 3441).

*Rhinella margaritifera*.— ECUADOR: PROVINCIA ORELLANA: Parque Nacional Yasuní, Estación Científica Yasuní (0.6772°S, 76.4012°W), 230 m (QCAZ 8415, 17736, 17740, 41011); Parque Nacional Yasuní, Bloque 31 (0.942°S, 75.905°W), (QCAZ 11909); Parque Nacional Yasuní, Rio Yasuní (0.9248°S, 75.9152°W), 206 m (QCAZ 11940); Parque Nacional Yasuní, Via Pompeya-Iro (0.6536°S, 76.4536°W), 287 m (QCAZ 17216, 17329, 43011, 22401); Parque Nacional Yasuní, Apaika (0.8656°S, 75.9245°W), (QCAZ 33545); Estación Biológica Tiputini (0.0639°S, 76.1493°W), 250 m (QCAZ 10207); Nuevo Rocafuerte (0.8967°S, 75.437°W),186 m (QCAZ 39466); Añangu (0.5249°S, 76.3844°W), 255 m (QCAZ 43952–953); Chiroisla (0.58°S, 75.9177°W), 207 m (QCAZ 44318–319; Huiririma (0.7116°S, 75.6239°W), 194 m (QCAZ 44563–565). PROVINCIA PASTAZA: Río Bobonaza (1.8056°S, 77.3313°W), 250 m (QCAZ 10650); Kapawi Lodge (2.5387 °S, 76.8583°W), 239 m (QCAZ 25476, 25488–489); Pomona (1.625°S, 77.9072°W), 846 m (QCAZ 25631). PROVINCIA SUCUMBIOS: Reserva Limoncocha (0.4062°S, 76.6195°W), 261 m (QCAZ 43104, 43108); Pañacocha (0.4712°S, 76.0667°W), 255 m (QCAZ 44098–099). PROVINCIA NAPO: Reserva Yachana (0.8333°S, 77.1667 °W), 350 m (**QCAZ 42269**); Cascada de San Rafael (0.1036°S, 77.5808°W), 1300 m (QCAZ 31708). PROVINCIA MORONA SANTIAGO: Plan de Milagro (3.0011 °S, 78.5052°W), 1950 m (QCAZ 48242).
